# Altered Env conformational dynamics as a mechanism of resistance to peptide-triazole HIV-1 inactivators

**DOI:** 10.1186/s12977-021-00575-z

**Published:** 2021-10-09

**Authors:** Shiyu Zhang, Andrew P. Holmes, Alexej Dick, Adel A. Rashad, Lucía Enríquez Rodríguez, Gabriela A. Canziani, Michael J. Root, Irwin M. Chaiken

**Affiliations:** 1 Department of Biochemistry and Molecular Biology, Drexel University College of Medicine, Philadelphia, PA USA; 2 School of Biomedical Engineering, Science and Health Systems, Drexel University, Philadelphia, PA USA; 3 Department of Microbiology and Immunology, Drexel University College of Medicine, Philadelphia, PA USA; 4 Faculty of Experimental Science, Francisco de Vitoria University, Madrid, Spain; 5 Department of Microbial Infection and Immunity, The Ohio State University College of Medicine, OH Columbus, USA

**Keywords:** HIV-1, Envelope glycoproteins, Entry inhibitor, Resistance mechanism, gp120 shedding, Virus escape, Macrocyclic peptide, Surface plasmon resonance (SPR), Isothermal titration calorimetry (ITC), Conformational dynamics

## Abstract

**Background:**

We previously developed drug-like peptide triazoles (PTs) that target HIV-1 Envelope (Env) gp120, potently inhibit viral entry, and irreversibly inactivate virions. Here, we investigated potential mechanisms of viral escape from this promising class of HIV-1 entry inhibitors.

**Results:**

HIV-1 resistance to cyclic (AAR029b) and linear (KR13) PTs was obtained by dose escalation in viral passaging experiments. High-level resistance for both inhibitors developed slowly (relative to escape from gp41-targeted C-peptide inhibitor C37) by acquiring mutations in gp120 both within (Val255) and distant to (Ser143) the putative PT binding site. The similarity in the resistance profiles for AAR029b and KR13 suggests that the shared IXW pharmacophore provided the primary pressure for HIV-1 escape. In single-round infectivity studies employing recombinant virus, V255I/S143N double escape mutants reduced PT antiviral potency by 150- to 3900-fold. Curiously, the combined mutations had a much smaller impact on PT binding affinity for monomeric gp120 (four to ninefold). This binding disruption was entirely due to the V255I mutation, which generated few steric clashes with PT in molecular docking. However, this minor effect on PT affinity belied large, offsetting changes to association enthalpy and entropy. The escape mutations had negligible effect on CD4 binding and utilization during entry, but significantly altered both binding thermodynamics and inhibitory potency of the conformationally-specific, anti-CD4i antibody 17b. Moreover, the escape mutations substantially decreased gp120 shedding induced by either soluble CD4 or AAR029b.

**Conclusions:**

Together, the data suggest that the escape mutations significantly modified the energetic landscape of Env’s prefusogenic state, altering conformational dynamics to hinder PT-induced irreversible inactivation of Env. This work therein reveals a unique mode of virus escape for HIV-1, namely, resistance by altering the intrinsic conformational dynamics of the Env trimer.

**Supplementary Information:**

The online version contains supplementary material available at 10.1186/s12977-021-00575-z.

## Background

HIV/AIDS remains a global health problem, with over 38 million people currently infected with the virus and 1.7 million new infections annually [1]. As neither a vaccine nor a cure is currently available, controlling infection requires continual administration of antiretroviral therapeutics. However, virus escape for many first- and second-line therapies is globally on the rise [2, 3] leading to a continual need for new therapeutic options. The Envelope glycoprotein (Env) is the only HIV-encoded protein on the virion surface and serves as the primary determinant of cellular tropism and viral entry. As such, Env represents an attractive target for both pre- and post-exposure prophylaxis. Nonetheless, only four FDA-approved HIV-1 entry inhibitors are on the market (Maraviroc, a CCR5 antagonist; Enfuvirtide, a fusion inhibitor; Fostemsavir, viral attachment inhibitor; and Ibalizumab, an anti-CD4 monoclonal antibody post-attachment inhibitor), and these are primarily employed for salvage therapy due to usage limitations [4, 5]. Env targeting remains an important opportunity to develop new strategies for HIV-1 intervention.

HIV-1 Env is a metastable and highly dynamic trimeric glycoprotein complex consisting of three surface gp120 subunits and three transmembrane gp41 subunits. In the metastable prefusogenic-state, gp120 binds to the host cell CD4 receptor located on T-lymphocytes and cells of the monocyte/macrophage lineage [6]. CD4 binding induces conformational changes throughout Env that expose and stabilize gp120 epitopes vital for binding to a chemokine co-receptor, either CCR5 or CXCR4. Binding to the co-receptor, in turn, triggers significant structural rearrangements in gp41 that ultimately lead to the fusion of viral and cellular membranes and delivery of viral capsid into the cytoplasm [7–10].

We have speculated that the intrinsic metastability of Env should make it vulnerable to agents that can induce irreversible conformational changes in the absence of target membranes receptors, thereby inactivating the Env complex. For instance, in some Env variants, soluble CD4 (sCD4) treatment promotes rapid gp120 shedding and concomitant gp41 refolding on the viral surface [11–13]. We have previously identified peptide triazoles (PTs) that bind to gp120, destabilize the Env complex and permanently inactivate the virus before cell encounter [14–20]. PTs appear to bind to a two-site cavity within gp120, as mapped by mutagenesis and computational docking [15, 19]. The first site is a CD4 binding-site subcavity occupied by Ser375; the second site is a nearby subcavity filled by Trp112 [15]. PT occupancy of this specific two-subcavity binding site results in large-scale changes in Env structure and function, including receptor binding blockade, gp120 shedding and altered gp41 exposure [14, 15, 21, 22]. Docking in the two-subcavity binding site has led to the development of macrocyclic PTs, denoted cPTs, that retain the functional signature of the PT class of Env inactivators and at the same time exhibit a striking proteolytic stability important for a potential drug-like candidate [15, 21, 22].

Given their potential utility as HIV-1 inactivators, it is important to determine how the virus could escape from PT antiviral activity. Here, we used a dose-escalation strategy to determine resistance pathways for both a cyclic PT (AAR029b) and a linear PT (KR13) and investigated the escape mechanism. Both PTs gave rise to similar resistance profiles, with mutations both within and distant to the canonical PT binding site. The result suggests that blocking the shared IXW pharmacophore was the principal factor driving viral escape. Through analyses of structure–activity relationships (SAR), cross-resistance profiles and ligand-induced gp120 shedding, we concluded that direct binding disruption played only a minor role in the mechanism of PT resistance. Rather, the escape mutations appeared to alter the conformational equilibria and dynamics of prefusogenic Env in a manner that stabilized trimer integrity and impeded PT-triggered viral inactivation.

## Results

### Generating HIV-1 resistance to peptide triazole inhibitors

**Fig. 1:**
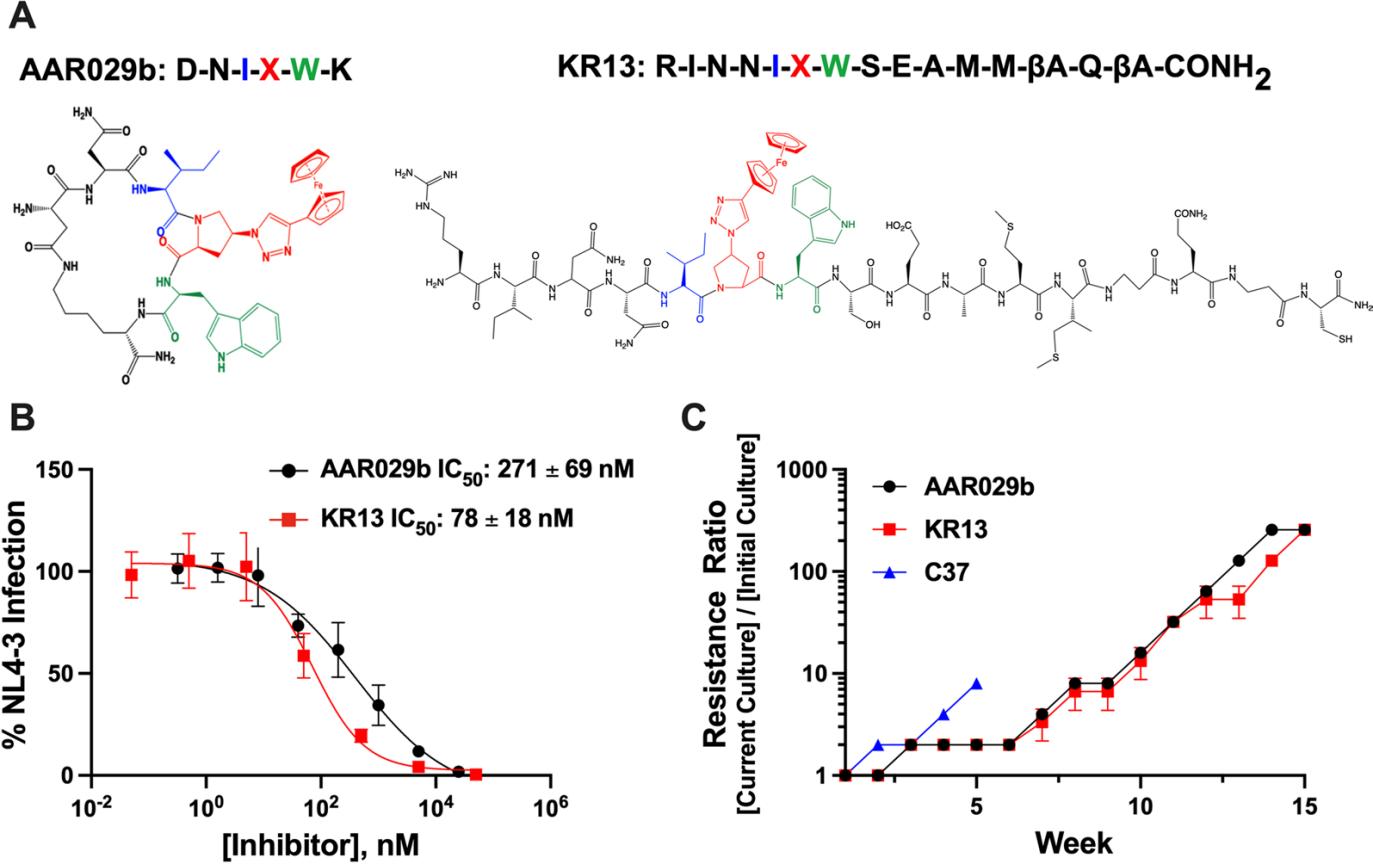
Generating resistance to peptide triazole inhibitors of HIV-1 entry. **A** Structures of macrocyclic PT AAR029b and linear PT thiol KR13. The two PTs share the same pharmacophore, I-X-W, where X represents an azidoproline with a ferrocene group. **B** PT inhibitory titrations against replicating HIV-1_NL4-3_. Viruses were added to CEM-GFP cells in the presence or absence of inhibitor, and the viral content in each culture was measured six days later by p24 ELISA. Data represent the mean and standard deviation of three independent experiments. **C** Dose-escalation profiles of KR13 (red), AAR029b (black) and C37 (blue) used during virus passaging. Inhibitor concentration was initiated at half of the IC_50_ value and increased when the p24 levels in culture supernatant were comparable to the p24 levels of cultures propagated in the absence of inhibitor. The data are expressed as the ratio of inhibitor concentration in culture at a given week to the initial inhibitor concentration at week zero. C37 is a gp41-targeted fusion inhibitor with a well-defined escape profile for HIV-1_NL4-3_ and served as a positive control for these assays. Data represent mean and standard deviation from three independent cultures for each inhibitor

Two potent HIV-1 inhibitors were selected for escape analysis from two different peptide triazole families (Fig. 1A). AAR029b is a macrocyclic peptide triazole (cPT) with drug-like properties such as proteolytic stability [21]. KR13 is a linear peptide triazole thiol (PTT) that is able to induce cell-free virolysis [16, 17, 20, 23]. AAR029b and KR13 induce gp120 shedding from BaL.01 viruses with EC_50_ values of 315 nM and 32 nM, respectively [16, 21]. In turn, shedding leads to irreversible inactivation of HIV virions. To define initial PT concentrations necessary for the dose-escalation protocol, we first determined the potencies of each of these inhibitors against replication-competent wild type HIV-1_NL4-3_ using CEM-GFP target cells. AAR029b and KR13 demonstrated antiviral potencies (IC_50_) of 271 nM and 78 nM, respectively (Fig. 1B) correlating well with previously published results using recombinant HIV-1 pseudotyped with BaL.01 and JR-FL Env on HOS-CCR5 cells [16, 17, 20].

Resistant HIV-1_NL4-3_ was generated by propagating viral cultures in the presence of increasing concentrations of PTs and tracking viral recovery by measuring p24 content in the supernatant. Specifically, inhibitor concentrations were raised only after viral titers were comparable (within threefold) to those from cultures propagated without inhibitor. Dose-escalation plots showed that resistance began to emerge by the sixth week after continuous treatment with AAR029b or KR13 and increased steadily during viral propagation (Fig. 1C). By comparison, treatment with control fusion inhibitor C37, which blocks structural changes in gp41, showed much more rapid emergence of resistance [24]. Viral cultures were propagated for four months and achieved final inhibitor concentrations of 125 μM for AAR029b and 16 μM for KR13, approximately 250-fold greater than their original concentrations at the start of viral propagation. The decision to stop cultures at that time was made to avoid interference from cellular cytotoxicity that could arise at higher inhibitor concentrations (Additional file 1: Figure S1).

### Genetic signatures of PT resistance

**Fig. 2:**
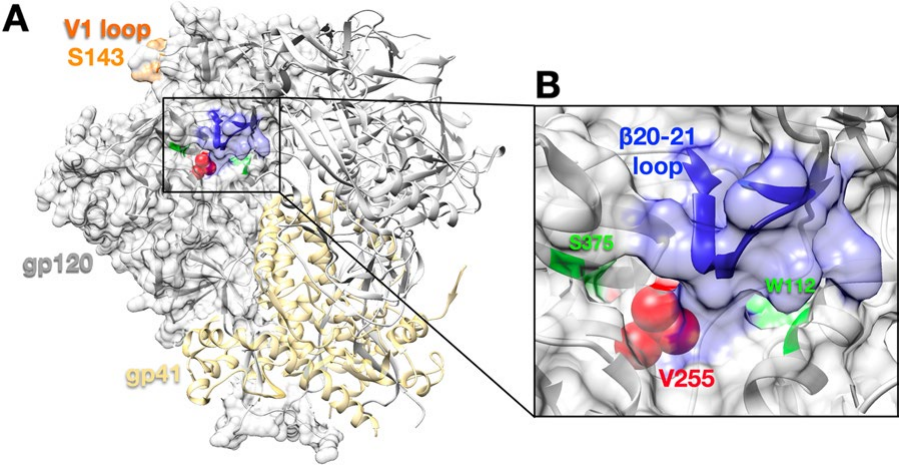
Location of Ser143 and Val255 in the prefusogenic structure of a Clade B HIV-1 Env trimer (PDB: 5FUU) [9]. **A** Env subunits gp120 (gray) and gp41 (pale yellow) are shown in cartoon representation with surface rendering of one gp120 subunit. Ser143 is located in the flexible V1 loop (orange) on the apical surface while Val255 (red) is located in a cavity that forms part of the putative PT binding pocket. **B** Enlargement of the PT binding cavity showing the positions of Trp 112 and Ser375 (green) [15, 19] that define the two subcavities of the PT binding site and the β20-21 loop (blue) that plays a critical role in Env activation

Env sequences from replicating viruses were obtained from integrated proviral DNA at 6, 10, and 15 weeks after the start of the dose-escalation protocol (Additional file 1: Figure S2). These timepoints were selected to provide sequences from the beginning, intermediate, and final stages of viral escape, when inhibitor concentrations were 2-, 16- and ~ 250-fold greater than starting IC_50_ values. Sequence alignments at the 6-week timepoint suggested that early resistance to both inhibitors was associated mainly with a single mutation, S143N (Table 1). This site is located in the gp120 variable loop region 1 at the apex of the Env trimer (Fig. 2), and the mutation disrupts potential N-linked glycosylation at position 141. At week 10, mutations at Val255 were observed in all peptide-treated cultures. Val255 is located within a β-turn between the inner and outer domains of gp120 subunit [25–27] and is identically conserved in 97% of Env sequences across all HIV-1 clades (total 4736, Los Alamos National Laboratories Database, November 2020) (Fig. 2). The V255I mutation was observed in all AAR029b cultures and two out of three KR13 cultures; HIV-1 in the third KR13 culture developed the V255T mutation (Table 1). Of note, Ile255 is observed in 2.7% of Env isolates in the database. Other mutations arose sporadically or transiently (e.g., V87A, R476K) but were not sustained in all cultures throughout the time course of viral propagation.

**Table 1:**
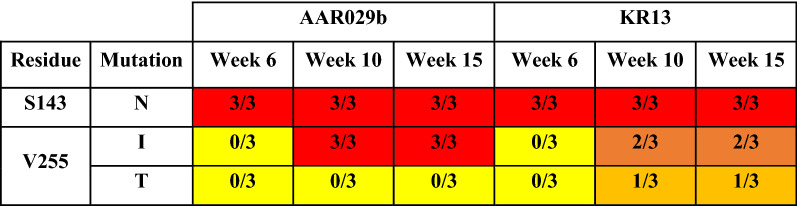
Summary of PT escape mutations identified from passaging studies

Proviral DNA sequences were obtained from three independent viral cultures at weeks 6, 10 and 15 of viral propagation and aligned to identify prominent mutations in Env (Additional file 1: Figure S2). The boxes show the fraction of cultures displaying the designated mutations and are color coded as follows: red: 3/3; dark orange: 2/3; light orange: 1/3; yellow: 0/3

### Impact of S143N and V255I/T mutations on PT inhibition

**Fig. 3:**
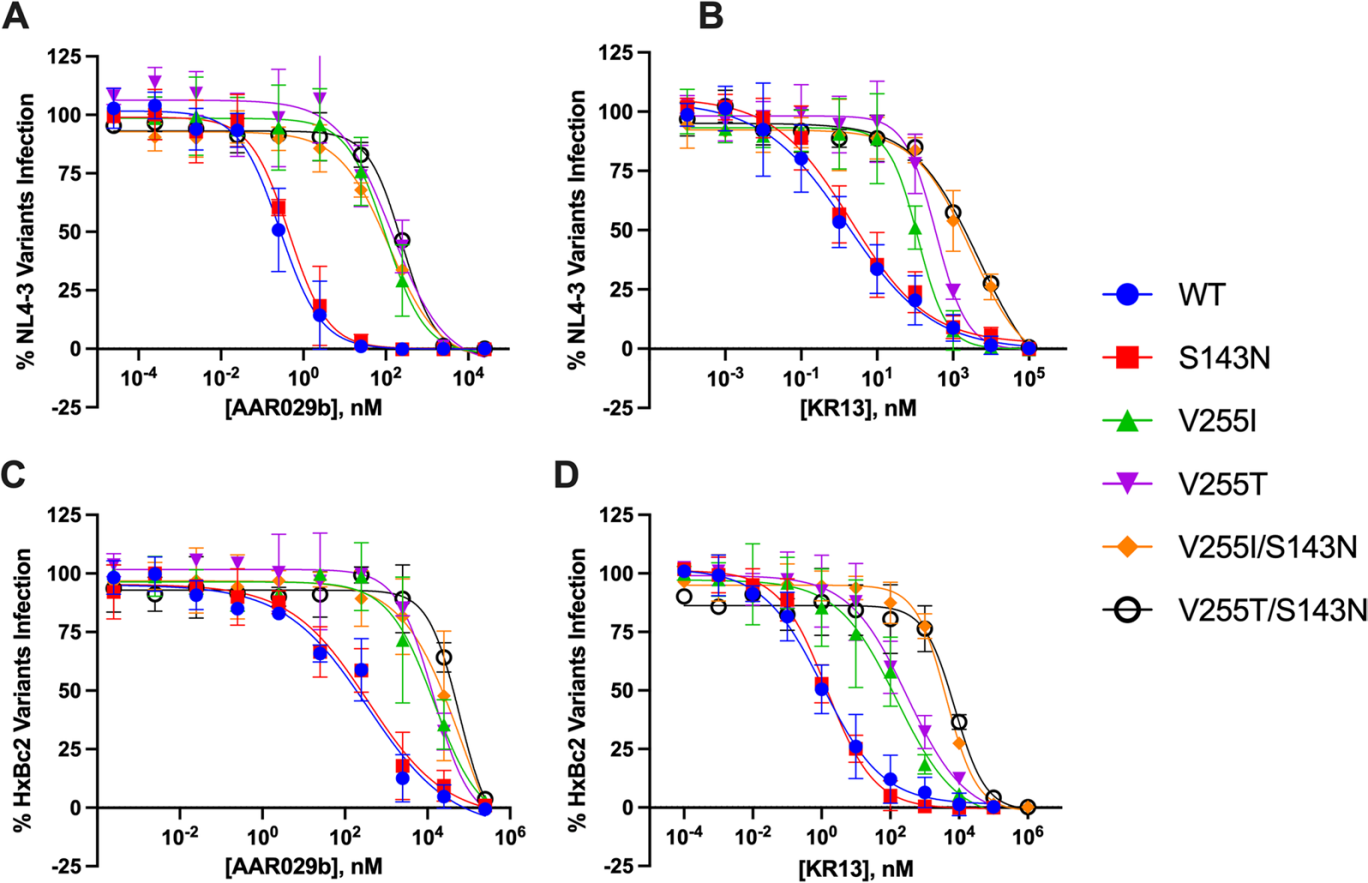
Peptide triazole inhibition of HIV-1 pseudotyped with wild type or resistant Envs. The activities of AAR029b (**A**, **C**) and KR13 (**B**, **D**) were measured against Envs from NL4-3 (**A**, **B**) and HxBc2 strains (**C**, **D**) that contained the following mutations: S143N (red), V255I (green) V255T (purple), V255I/S143N (orange), V255T/S143N (black, open symbol). The wild type Env titration is shown in blue. Data represent mean and standard deviation of five independent experiments performed in triplicate. IC_50_ values are reported in Table 2

Mutations at Ser143 and Val255 were reproduced in recombinant plasmids encoding HIV-1 Env (NL4-3 and HxBc2), and tested against inhibition by AAR029b and KR13 in single-round viral infectivity assays (Fig. 3 and Table 2). As a single mutation, S143N had minimal (less than 2.5-fold) impact on Env sensitivity to linear and cyclic PTs, consistent with its appearance at the earliest timepoints of selection. By contrast, the V255I and V255T mutations, which first appeared at ten weeks of viral culture, exerted a 50- to 370-fold disruption in potency for both PTs. Curiously, dual substitutions at both Ser143 and Val255 had a differential impact on the potencies of the two PTs. Compared to Val255 mutations alone, combining S143N and V255I/T further disrupted the potency of linear PT KR13 15- to 30-fold, but had only a minor (two to threefold) effect on cyclic PT AAR029b. The data indicate that the major determinant of PT resistance is residue position 255. The impact of the S143N mutation was dependent on both the amino acid at residue 255 and the type (cyclic versus linear) of PT. The results suggest a previously unappreciated connection between two disparate sites on Env**.**

**Table 2:** Inhibitory properties of AAR029b and KR13 against HIV-1 pseudotyped with wild type and resistance Env variants

	AAR029b IC_50_ (nM) (fold-change relative to WT)		KR13 IC_50_ (nM) (fold-change relative to WT)	
	NL4-3	HxBc2	NL4-3	HxBc2
Wild type	250 ± 90 (1)	230 ± 130 (1)	2.0 ± 0.56 (1)	1.2 ± 0.17 (1)
S143N	380 ± 20 (1.5)	540 ± 50 (2.3)	3.5 ± 1.5 (1.8)	1.4 ± 0.24 (1.2)
V255I	7.6 × 10^4^ ± 1.6 × 10^4^ (300)	1.4 × 10^4^ ± 810 (50)	120 ± 18 (62)	91 ± 23 (77)
V255T	9.1 × 10^4^ ± 3.9 × 10^4^ (370)	1.5 × 10^4^ ± 1.5 × 10^4^ (62)	360 ± 35 (180)	330 ± 70 (280)
V255I/S143N	2.0 × 10^5^ ± 2.6 × 10^4^ (790)	3.4 × 10^4^ ± 2.9 × 10^4^ (150)	3700 ± 560 (1900)	4600 ± 510 (3900)
V255T/S143N	2.4 × 10^5^ ± 2.7 × 10^4^ (980)	3.7 × 10^4^ ± 1.8 × 10^4^ (160)	6200 ± 320 (3100)	7100 ± 1100 (6000)

### Impact of S143N and V255I/T mutations on the gp120-PT interaction

**Fig. 4:**
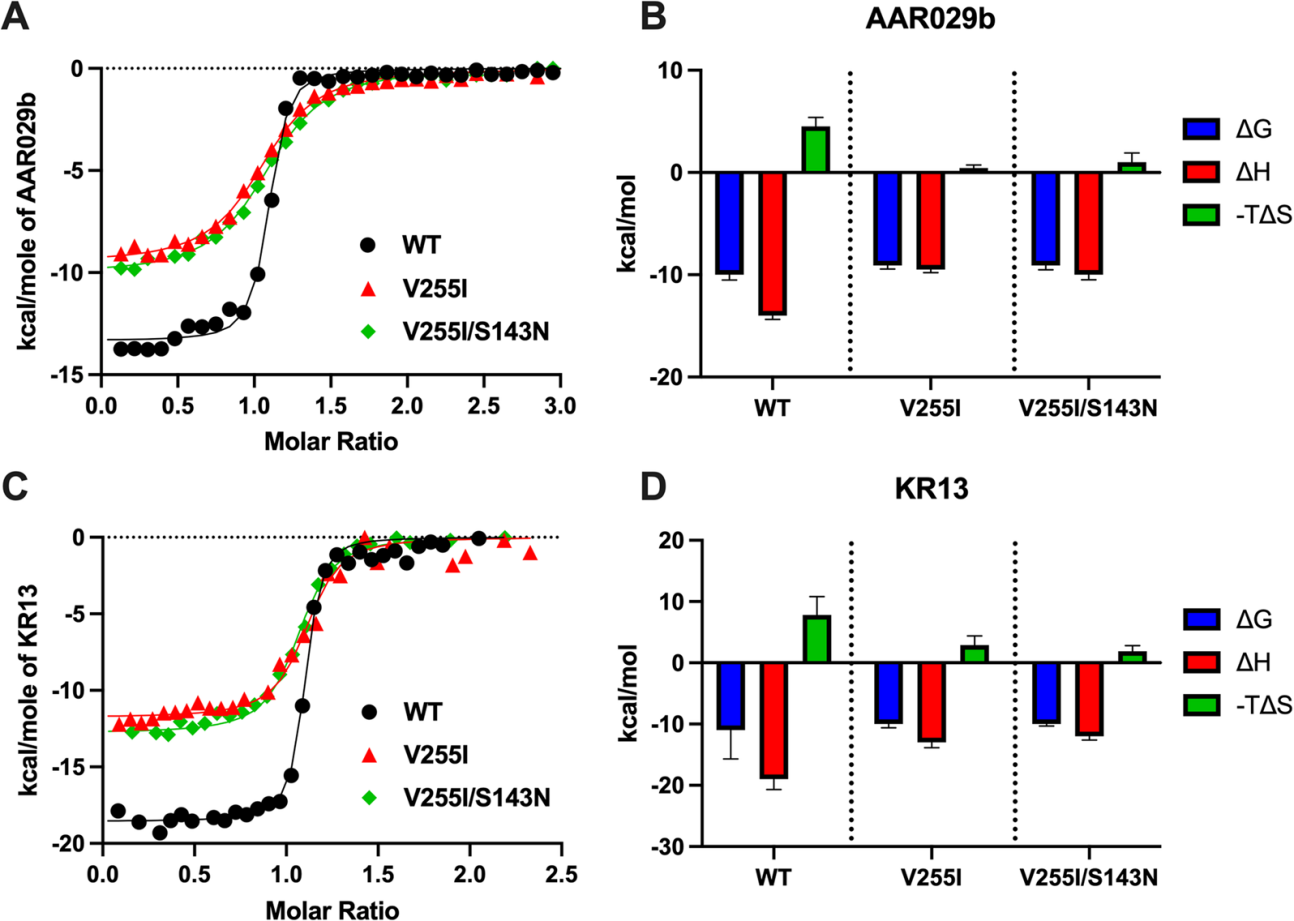
Thermodynamic dissection of AAR029b and KR13 binding to wild type and resistant gp120 by isothermal titration calorimetry. **A** and **C** Binding isotherms of AAR029b (**A**) and KR13 (**C**) to wild type (black), V255I (red) and V255I/S143N (green) monomeric gp120 variants. Heats were obtained at 25 °C by injection of 8 μL of PT (100 μM) into a sample cell of a VP-ITC containing 1.4 mL recombinant HxBc2 gp120 (6–8 μM). Solid lines represent fits to a single site binding model. **B** and **D** The thermodynamic parameters free energy (blue), enthalpy (red) and entropy (green) extracted from the fits to the AAR029b (**B**) and KR13 (**D**) titrations in **A** and **C**. The data shown in **A** and **C** are derived from a single binding experiment for each condition. The data shown in **B** and **D** and summarized in Table 3 are the mean and range of mean of two independent experiments

To ascertain the mechanism of resistance, we first investigated how the V255I and V255I/S143N mutations influenced the binding interactions of PTs with monomeric gp120 (HxBc2 strain). Using isothermal titration calorimetry (ITC), we found that both the cyclic and linear PTs bound to wild type gp120 with similar low- to mid-nanomolar affinity, and that association was enthalpically favorable but entropically unfavorable (Fig. 4 and Table 3). As expected, the PT-to-gp120 binding stoichiometry (n) was approximately 1. The V255I mutation decreased the affinities of both inhibitors, ninefold for the cyclic PT AAR029b (K_D_ value shift from 25 to 220 nM) and fourfold for the linear PT KR13 (16 nM to 59 nM). Adding the S143N mutation to the V255I mutant gp120 background had little to no further effect on binding strength. Thermodynamically, the V255I substitution made PT association significantly less enthalpically favorable, consistent with fewer bonding interactions. Unexpectedly, PT interaction with the mutant gp120 variant was also found to be less entropically unfavorable, pointing to an impact of the V255I substitution on gp120 conformational flexibility. The absolute impact on enthalpy (ΔH) and entropy (TΔS) was nearly identical (3.5–4.5 kcal/mol for AAR029b, 5–7 kcal/mol for KR13), almost canceling out their individual effects on interaction free energy (ΔG) (Fig. 4B, D).

**Table 3:** Thermodynamic properties of PT binding to WT and mutant gp120 variants (HxBc2 strain)

	ITC^a^				
	K_D_ (nM)	ΔH (kcal/mol)	ΔG (kcal/mol)	−TΔS (kcal/mol)	n
**AAR029b**					
WT	25 ± 2.0	− 14 ± 0.37	− 10 ± 0.52	4.5 ± 0.90	1.1 ± 0.04
V255I	220 ± 2.0	− 9.5 ± 0.29	− 9.1 ± 0.33	0.43 ± 0.30	1.0 ± 0.01
V255I/S143N	230 ± 12	− 10 ± 0.49	− 9.1 ± 0.42	1.0 ± 0.91	1.1 ± 0.04
**KR13**					
WT	16 ± 1.4	− 19 ± 1.7	− 11 ± 4.7	7.8 ± 3.0	1.1 ± 0.01
V255I	59 ± 9.2	− 13 ± 0.86	− 10 ± 0.61	2.9 ± 1.5	1.0 ± 0.04
V255I/S143N	59 ± 0.60	− 12 ± 0.62	− 10 ± 0.31	1.9 ± 0.93	1.1 ± 0.08

^a^ITC data: mean of two replicates ± range of mean

**Fig. 5:**
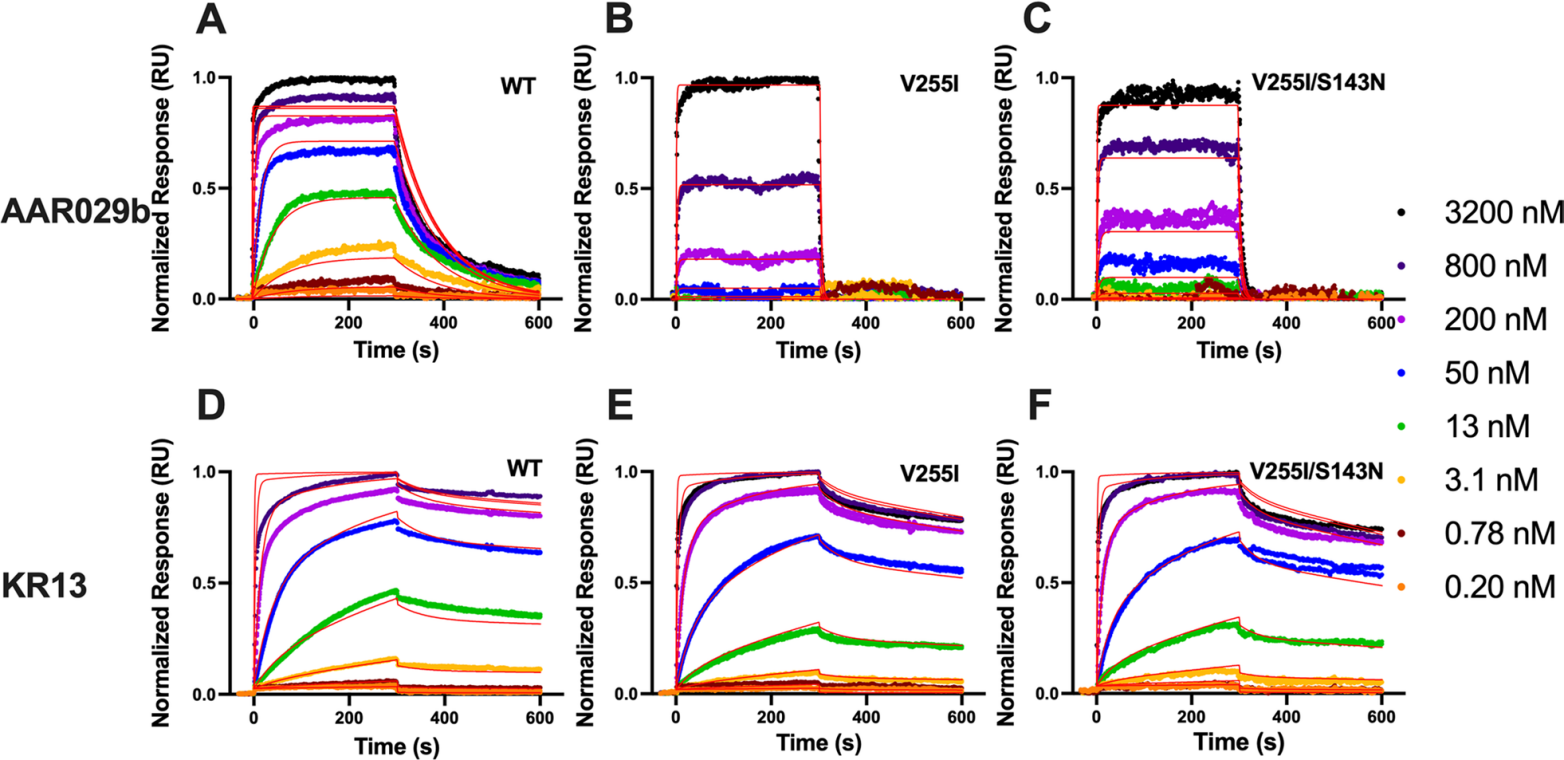
Kinetic analysis of AAR029b and KR13 binding to wild type and resistant gp120 by surface plasmon resonance. The Biacore 3000 sensorgrams depict the interaction of AAR029b (**A**–**C**) and KR13 (**D**–**F**) with wild type (**A** and **D**), V255I (**B** and **E**) and V255I/S143N (**C** and **F**) HxBc2 gp120 variants covalently attached to the sensor chip surface at 25 °C. The global fits to two replicates, as defined in Materials and Methods, are shown as red lines. The binding parameters derived are summarized in Table 4

In order to confirm effects of escape mutations on inhibitor binding, the kinetic profiles of gp120-PT interactions were measured using surface plasmon resonance (SPR) (Fig. 5 and Table 4). As seen with ITC, the V255I mutation decreased the binding affinity (K_D_) of AAR029b (31-fold), and subsequent addition of the S143N mutation only minimally disrupted binding further. The AAR029b affinity disruption resulted primarily from an elevated inhibitor dissociation rate, k_-1_, consistent with fewer bonding interactions. As reported previously, the KR13 binding curves did not follow simple bimolecular interaction kinetics, possibly due to the ability of the KR13 thiol group to induce disulfide reshuffling in Env [15]. To fit the fast and slow phases of the interaction, we applied a three-state equilibrium model with the following rate constants: k_1_ and k_-1_ reflecting inhibitor association and dissociation from Env; and k_2_ and k_-2_ reflecting inhibitor induced disulfide isomerization and de-isomerization (details in “Materials and Methods” and Additional file 1: Method S1). The escape mutations minimally impacted these rate constants, with the greatest effect (three to fourfold) observed for de-isomerization kinetics. As a result, the KR13 affinity was only modestly disrupted (four to sixfold). Overall, the SPR data were consistent with findings from ITC measurements, specifically that escape mutation V255I only modestly disrupted PT binding affinity, and the S143N mutation had minimal additional impact.

**Table 4:** Kinetic properties of PT binding to WT and mutant gp120 variants (HxBc2 strain)

	SPR				
	K_D_ (nM)	k_1_ (M^−1^ s^−1^) × 10^–5^	k_-1_ (s^−1^) × 10^3^	k_2_ (s^−1^) × 10^3^	k_-2_ (s^−1^) × 10^3^
**AAR029b**					
WT	7.8	11	8.3	–	–
V255I	240	5.4	130	–	–
V255I/S143N	300	3.6	110	–	–
**KR13**					
WT	1.1	2.9	9.6	7.0	0.25
V255I	4.8	2.3	14	8.4	0.72
V255I/S143N	6.5	2.8	18	9.0	1.0

### Mechanism of affinity disruption caused by the V255I/T escape mutations

**Fig. 6:**
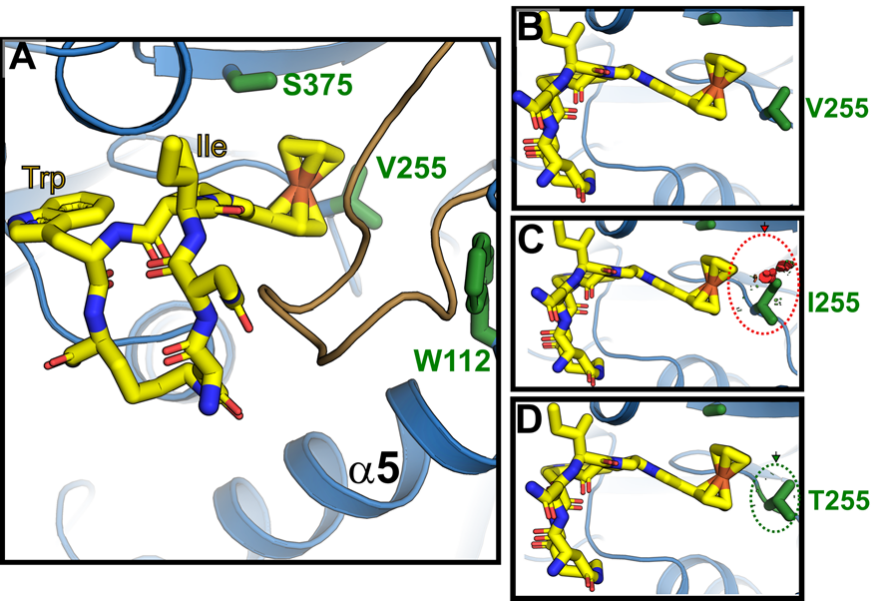
Flexible-docking models of AAR029b bound to wild type and mutant Env trimers. **A** Low energy pose of AAR029b docked into the putative PT binding site in a wild type HIV-1 Env (JR-FL strain, PDB: 5FUU[9]). The side chains of important subcavity residues Trp112 and Ser375, as well as Val255 identified in this study, are shown as green sticks. The β_20-21_ loop is highlighted in brown. **B**–**D** Steric clashing of the PT ferrocenyl group with the side chain at residue position 255. The side chains of Val (**B**), Ile (**C**) and Thr (**D**) are depicted in their most prominent conformers. The sizes of the red disks indicate the degree of clashing with the side chain with red (**C**) and green (**D**) dotted circles indicated

To evaluate how Val255 mutations might perturb PT binding, we performed molecular dynamics, flexible docking calculations of AAR029b binding to a model of a fully glycosylated Clade B Env trimer (JR-FL, PDB ID: 5FUU) [9]. With the wild type protein, the cyclic PT assumed low energy conformations that aligned well with previous computational and laboratory data using linear PTs (Fig. 6A, B) [15, 19]. The inhibitor inserted its pharmacophore, the indole side chain and ferrocene moiety, into two adjacent binding subsites on Env, one that contains residue Ser375 of the CD4 Phe43 binding pocket and another that contains residue Trp112, which is critical for Env structural rearrangements during HIV-1 entry [15] (Fig. 2). Of note, the ferrocene moiety adopts a conformation less than 4 Å from the γ carbons on the Val255 side chain with no steric clashing.

We carried out in silico mutagenesis of Val255 in the context of the AAR029b docked structure to identify potential clashes that would disrupt inhibitor binding. The V255I mutant gp120 largely tolerated inhibitor binding, with only a few problematic Ile rotamers showing clashes between the side-chain δ-methyl group and AAR029b ferrocene moiety (Fig. 6C). The modeling did not show the degree of steric clashing expected if the mutation substantially blocked gp120-PT bonding interactions, as implied by the large change in binding enthalpy measured in ITC. Interestingly, modeling of Thr at position 255 revealed even less steric clashing although the V255I and V255T mutations had similar impacts on PT resistance (Fig. 6D). Although it is possible that the Thr substitution could alter the hydrophobic packing of the PT ferrocene, neither substitution appeared to disrupt AAR029b interactions with gp120 in silico.

Overall, the computational modelling and interaction measurements poorly accounted for the observed resistance. The effects of the V255I and S143N mutations on PT affinity for monomeric gp120 were substantially less than the magnitude of their impact on PT potency (Tables 2, 3, 4). This discrepancy might reflect structural differences between the PT binding sites in gp120 monomers *versus* functional Env trimers. However, the paradoxical effect of the V255I mutation on interaction entropy pointed to additional mechanisms of resistance that involve changes in the dynamic properties of Env. Specifically, the entropic penalty to PT association decreases when Val255 is substituted with Ile, suggesting that the escape mutation alters gp120 conformational fluctuations. In the context of the Env trimer, the escape mutations might well impact the distribution among prefusogenic states, thereby altering the ability of PTs to induce allosteric changes critical to binding stabilization. Moreover, the escape mutations may affect post-binding, nonequilibrium conformational changes in Env trimers that binding measurements on gp120 monomers or molecular modeling employing largely static Env trimers do not replicate. Based on these considerations, we refocused our exploration of PT resistance on dissecting the functional impact of escape mutations on the Env glycoprotein itself.

### Impact of PT escape mutations on neutralization and binding of mAb 17b

**Fig. 7:**
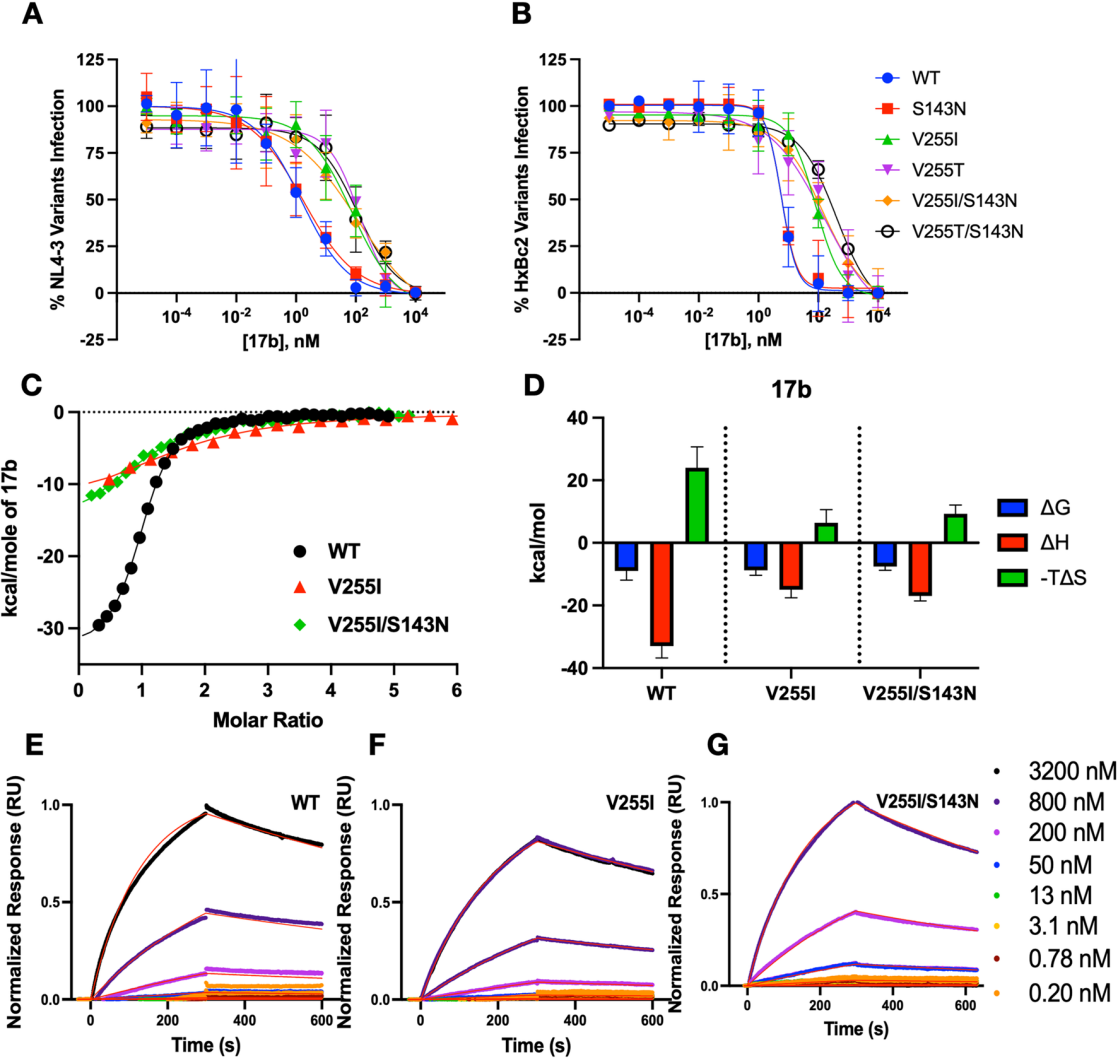
Effect of escape mutations on the neutralization and binding properties of mAb 17b. **A**–**B** Neutralization activity of 17b IgG against HIV-1 pseudotyped with wild type and resistant Envs from strains NL4-3 (A) and HxBc2 (B). Data reflect the mean and standard deviation of five independent experiments. **C** Binding isotherms of 17b to wild type, V255I and V255I/S143N monomeric gp120 variants obtained by ITC. Solid lines represent fits to a single-site binding model. Data represent mean and range of mean of two independent experiments performed. **D** Thermodynamic parameters free energy, enthalpy and entropy extracted from the fits in **C**. The data in **C** and **D** are derived from a single binding experiment for each condition. The titrations were repeated with quantitatively similar results. **E**–**G** Biacore 3000 sensorgrams depicting 17b interaction with immobilized monomeric gp120: WT (**E**), V255I (**F**) and V255I/S143N (**G**). The global fits to two replicates, as defined in Materials and Methods, are shown as red lines. The binding parameters derived are summarized in Table 5

To explore whether PT resistance might reflect an altered conformational landscape of Env, we investigated the impact of escape mutations on antiviral neutralization by mAb 17b. The antibody binds to a four-stranded β-sheet in gp120 that forms upon CD4 binding [28]. This structure, called the bridging sheet, is a component of the chemokine-receptor binding site on Env and, thus, its formation plays a critical role in HIV-1 entry [29]. A loop connecting two of the β strands (β20–β21) has been identified as a master regulator of Env conformational transitions [30–35]. This loop is juxtaposed to Val255 in both the prefusogenic (Figs. 2 and 6A) and fully activated states of gp120 [8, 10]. Given the proximity of the loop to the PT binding pocket, we reasoned that 17b neutralization might be particularly sensitive to alterations in Env dynamics induced by PT escape mutations. Indeed, the V255I and V255T substitutions reduced 17b potency more than tenfold against Env HxBc2 and more than 40-fold against Env NL4-3 (Fig. 7A, B and Table 5). As observed with PT inhibition, the S143N substitution by itself did not impact 17b neutralization. However, introducing the S143 substitution into an Env already containing a V255I/T mutation further reduced 17b potency 2.5- to 4-fold. Because neither Val255 nor Ser143 are in the binding epitope for mAb 17b, mutations at these sites must mediate their effect on antibody neutralization indirectly, possibly by altering spatiotemporal properties of epitope exposure.

**Table 5:** Neutralization and binding properties of mAb 17b against WT and resistant Env

	Neutralization IC_50_ (nM) (fold-change relative to WT)		ITC^a^					SPR		
	NL4-3	HxBc2	K_D_ (nM)	ΔH (kcal/mol)	ΔG (kcal/mol)	−TΔS (kcal/mol)	N	K_D_ (nM)	k_1_ (M^−1^ s^−1^) × 10^–3^	k_-1_ (s^−1^) × 10^4^
Wild type	1.3 ± 0.39 (1)	6.1 ± 3.2 (1)	280 ± 5.0	− 33 ± 3.8	− 9.0 ± 2.9	24 ± 6.7	1.0 ± 0.03	130	4.2	5.4
S143N	2.0 ± 0.50 (1.6)	6.2 ± 1.7 (1.0)	−					−		
V255I	55 ± 29 (44)	78 ± 42 (13)	900 ± 20	− 15 ± 2.6	− 8.8 ± 1.6	6.4 ± 4.2	1.5 ± 0.09	500	1.5	7.4
V255T	110 ± 26 (89)	120 ± 100 (19)	–					–		
V255I/S143N	250 ± 190 (200)	250 ± 110 (40)	1000 ± 20	− 17 ± 1.6	− 7.6 ± 1.2	9.3 ± 2.8	1.1 ± 0.06	530	1.8	9.4
V255T/S143N	270 ± 89 (220)	350 ± 85 (57)	–					–		

^a^ITC data: mean of two replicates ± range of mean

To decipher the mechanism of 17b cross-resistance, we investigated how PT escape mutations affected antibody binding to monomeric gp120 using ITC and SPR (Fig. 7C–G and Table 5). The V255I substitution exerted only a modest effect on binding affinity (three to fourfold), and adding the S143N substitution to this background did not disrupt binding further. The small (less than 1 kcal/mol) impact of the V255I mutation on interaction free energy (ΔG) belied significant, compensatory changes in interaction enthalpy (ΔH) and entropy (TΔS). Association was more than 16 kcal/mol less favorable in enthalpy, but more than 14 kcal/mol more favorable in entropy. This same pattern in binding thermodynamics was qualitatively observed in PT interaction experiments (compare Tables 3 and 5). The qualitative similarities in both the inhibition and interaction thermodynamic data for PTs and mAb 17b imply that the two inhibitor classes share the same mechanism of resistance. As PTs and mAb 17b have widely disparate physiochemical properties and interact with different binding sites on Env, the results strongly suggest that the escape mutations exert their binding effects by altering Env conformational dynamics. Interestingly, SPR analysis revealed that the escape mutations primarily altered the association rate of 17b, in contrast to their primary impact on dissociation rate for PTs. We attribute this difference in kinetic consequences of escape mutations to the ability of PTs to interact with multiple prefusogenic Env conformations while 17b is restricted to binding the CD4-induced state (see “Discussion”).

### Impact of PT escape mutations on CD4 interactions with Env

**Fig. 8:**
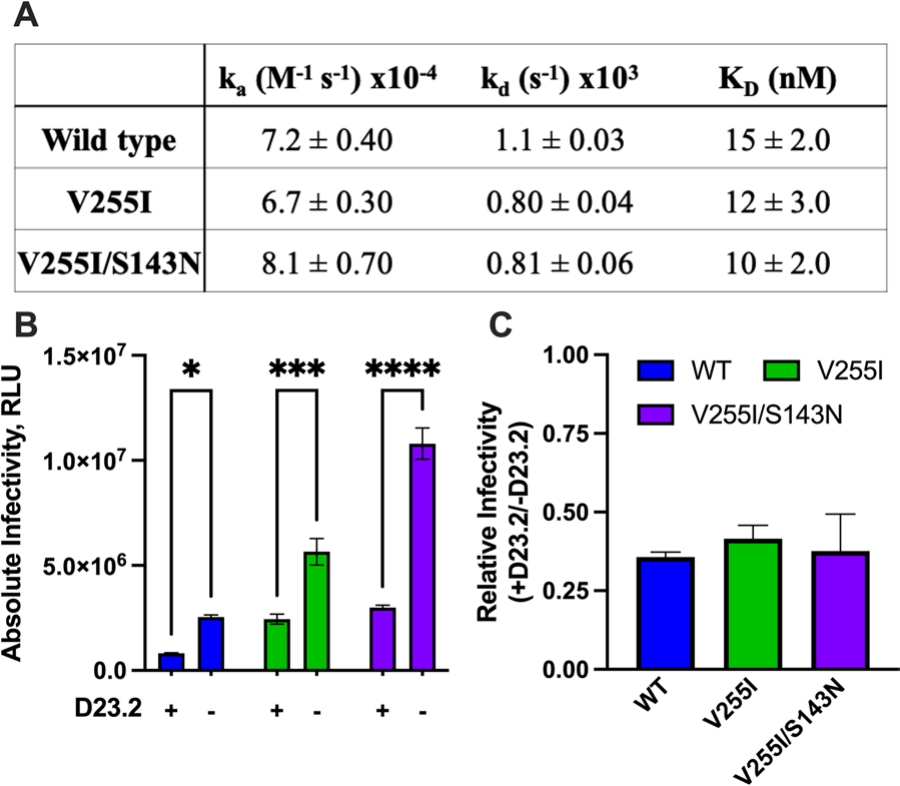
Effect of PT escape mutations on CD4 binding and utilization during entry. **A** Binding paramerters derived from the interaction of sCD4 with WT, V255I and V255I/S143N monomeric gp120 variants as measured by surface plasmon resonance (sensorgrams shown in Additional file 1: Figure S3). **B** Infectivity of HIV-1 pseudotyped with wild type and resistant Env variants in the absence and presence of CD4 antagonist D23.2 (11 nM). The significance of data was determined by two-way ANOVA in GraphPad Prism 9 (*p < 0.05, ***p < 0.0005, ****p < 0.0001). **C** Relative infectivities (normalized data from B) to illustrate the impact of escape mutations on D23.2 sensitivity. Data represent the mean and standard error of three independent experiments done in quadruplicate. There was no significant difference between groups as assessed by one-way ANOVA in GraphPad Prism 9

The effects of PT escape mutations on mAb 17b neutralization and binding led us to question whether they influenced CD4 interaction and utilization during entry. We first assessed binding of soluble CD4 (sCD4) to wild type and mutant gp120 by SPR. Neither the single V255I substitution nor the dual V255I/S143N substitution significantly perturbed sCD4 binding affinity or kinetics (Fig. 8A and Additional file 1: Figure S3). We next assessed CD4 utilization by the sensitivity of viral infection to DARPin D23.2, an antagonist that targets cellular CD4 and blocks its interaction with gp120 [36]. Alterations in Env that increase its dependence on cellular CD4 for viral entry (e.g., decreasing the Env-CD4 binding affinity or raising the required CD4 binding stoichiometry for entry) should result in an increased sensitivity to D23.2. Instead, we found that the wild type and mutant Envs were similarly inhibited at D23.2 concentrations near the IC_50_ value for wild type Env (Fig. 8B–C). Thus, PT escape mutations did not seem to substantially alter the structure or stability of gp120 states primarily responsible for binding CD4.

**Fig. 9:**
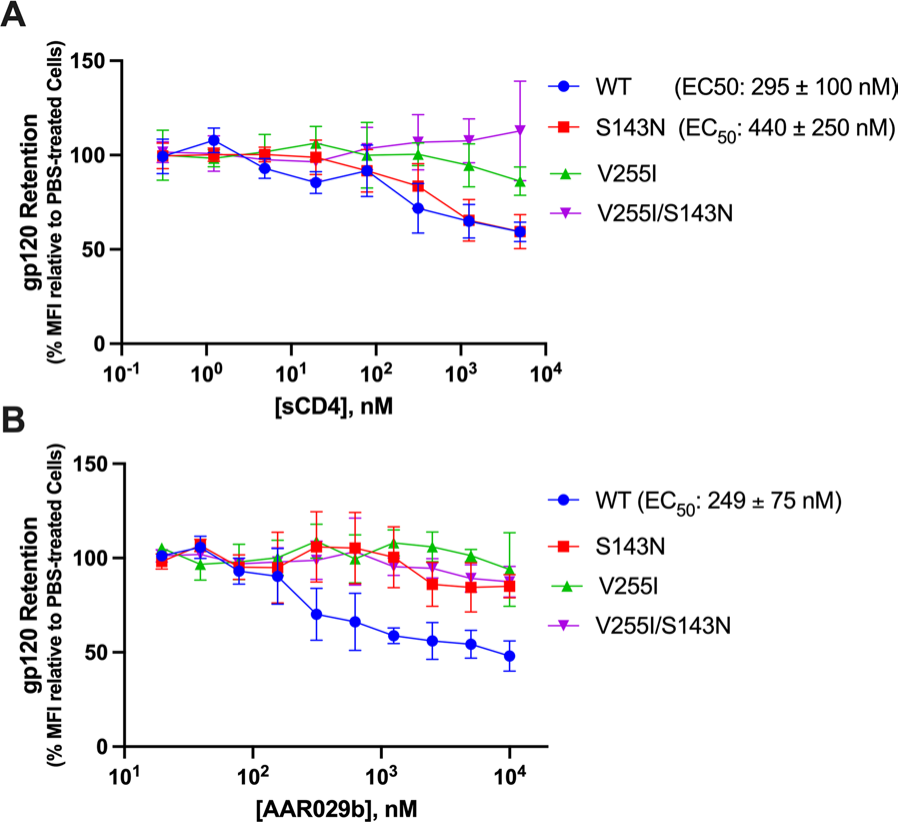
Impact of PT escape mutations on CD4- and AAR029b-induced gp120 shedding. **A** The percent of intact Env trimers remaining on HEK293T cells expressing wild type and resistant NL4-3 Env exposed to serial dilutions of sCD4 (5000 nM as the highest concentration) at 37 °C for 2 h. The measurements (mean fluorescence intensity) were obtained by flow cytometry using an anti-Env IgG (mAb 35O22) that recognizes a quaternary epitope at the gp120/gp41 interface. **B** As in **A**, except that the cells were exposed to serial dilutions of AAR029b (1 μM as the highest concentration). Data represent the mean and standard error of four independent experiments performed in quadruplicate

We next asked whether the PT escape mutations altered Env conformational transitions downstream of CD4 binding. Since PT association can induce gp120 shedding and 17b binds to a CD4-induced epitope, Env changes that destabilize post-CD4 binding states could lead to PT and 17b resistance without substantially altering CD4 interaction. We reasoned that such changes would manifest as resistance to sCD4-induced shedding. To test this conjecture, we incubated cells expressing wild type and mutant Env with increasing concentrations of sCD4 for two hours and then measured the remaining amount of the intact trimers by flow cytometry. Env variants with the V255I substitution or V255I/S143N dual substitution were dramatically less sensitive to sCD4-induced shedding (greater than tenfold) compared to wild type Env (Fig. 9A). The S143N substitution also showed a modest effect (twofold). These changes in sensitivity to sCD4-induced shedding qualitatively mirror the degree of PT and mAb 17b resistance exerted by the PT escape mutations. Not surprisingly, the escape mutations also dramatically reduced the PT concentration dependence to shedding (Fig. 9B). Here, not only were the V255I and V255I/S143N mutant Env variants more than tenfold resistant to PT-induced shedding, but the S143N mutant Env trimer showed only minimal disruption at 10 mM AAR029b, 50-fold higher than the EC_50_ value for wild type Env. Together, the binding, inhibition and shedding data suggest that PT resistance involves changes in the energetic landscape of the prefusogenic states that disrupt transitions induced by CD4 and PT binding.

## Discussion

Previous investigations of the PT class of HIV-1 Env inhibitors have led to derivation of small and proteolytically stable macrocyclic PTs (cPTs) that potently block virus-cell infection and trigger gp120 shedding and consequent irreversible virus inactivation before host cell encounter [14–23, 37–40]. The appealing antiviral functions and metabolic stability of the cPTs led us to investigate the nature of viral resistance to this inhibitor class. We used a dose-escalation approach to evaluate HIV-1 escape from a drug-like cPT, AAR029b [21], and compared it to escape from a linear PT, KR13 [16, 20]. The two inhibitors elicited escape mutations at the same two residues in gp120, one within the putative PT binding site (V255I/T) and the second outside the binding region on the Env apical surface (S143N) (Fig. 2). The mutations alone and in combination gave rise to the same resistance pattern for both PTs (Fig. 3). AAR029b and KR13 have the same pharmacophore (I-X-W, where X is the triazolePro residue with a ferrocenyl R group) (Fig. 1A), but KR13 possesses a terminal thiol group that can trigger an additional, unique functional response, virolysis [16, 21, 23]. Likely because this lytic process occurs over a higher concentration range than infection inhibition, KR13 shares the same resistance profile as AAR029b, with the main pressure driving escape being the pharmacophore common to both.

The dominant escape mutations selected in the presence of AAR029b and KR13 were found to have a far greater effect on PT inhibitory potency (Fig. 3 and Table 2) than on PT binding affinity (Figs. 4, 5 and Tables 3, 4). The relatively small affinity effect of the V255I/T substitutions, which was measured with gp120 monomers, is consistent with the docking analysis of AAR029b showing only partial clashes of the Ile and Thr side chains with the Ile-TriazolePro-Trp pharmacophore (Fig. 6). In addition, the S143N mutation exerts no impact on PT binding affinity, consistent with its location on the apical surface of Env distal to the inhibitor binding site. In fact, the effect of the S143N mutation on PT inhibition (especially for KR13) appears to depend on the specific amino acid at residue position 255, with a more significant disruption noted with Ile or Thr than with Val (Fig. 3 and Table 2). A significant caveat to these measurements is that ligand binding was measured with gp120 monomers and that escape mutations might exert more profound effects in the context of Env trimers. However, comparisons of functional inhibition and binding data so far suggest that direct disruption of the PT-Env interaction likely plays a relatively minor role in escape from PT inhibition.

Despite having only a small impact on binding affinity, the V255I substitution was found to exert profound and compensatory effects on association enthalpy and entropy. As indicated by the large reduction in enthalpic stabilization, the loss of bonding interactions, was almost entirely offset by the decreased entropic penalty to inhibitor association (Fig. 4 and Table 3). Moreover, these thermodynamic changes were manifest in an increased PT-gp120 dissociation rate constant, with minimal to no effect on the association rate constant. Together, the results suggest that the V255I mutation increases the conformational flexibility of PT-bound Env gp120. Thus, it appears that a major effect of the PT escape mutations is to alter the energy landscape of the Env trimer, thereby influencing transition equilibria and dynamics between different prefusogenic-state conformations.

Further support for the role of Env conformational dynamics in PT resistance comes from the impact of PT escape mutations on mAb 17b neutralization. Although spatially close, the PT and 17b binding sites do not overlap. Yet the pattern of 17b cross-resistance exerted by the S143N and V255I/T mutations is qualitatively identical to PT resistance, including the importance of residue 255 in determining the impact of the S143N substitution on antiviral potency (Tables 2 and 5). Similarly, the consequences of the escape mutations on 17b binding affinity and thermodynamics also mirror their effects on PT binding, including the large and offsetting differences in association enthalpy and entropy (Tables 3, 4, 5). The parallels in these inhibition and binding properties for PTs and 17b strongly argue that cross-resistance emerged mainly through altered Env conformational dynamics rather than direct binding disruption.

While PT escape mutations gave rise to 17b cross-resistance, they did not appear to disrupt CD4 binding affinity or utilization during HIV-1 entry (Fig. 8). The finding implies that these mutations destabilize conformational states downstream of CD4 binding. This conclusion is supported by two additional sets of data. First, the mutations reduce the association kinetics of mAb 17b (Table 5), which targets gp120 in the CD4-induced conformation after the formation of the bridging sheet. Second, the mutations render NL4-3 Env less sensitive to both CD4- and cPT AAR029b-triggered gp120 shedding (Fig. 9). A caveat is that gp120 shedding effects could be strain-dependent. Nonetheless, taken together, the pseudovirus results obtained so far suggest that inhibitor escape is at least partially achieved by an altered energetic landscape that enables Env to resist PT-induced irreversible inactivation.

**Fig. 10:**
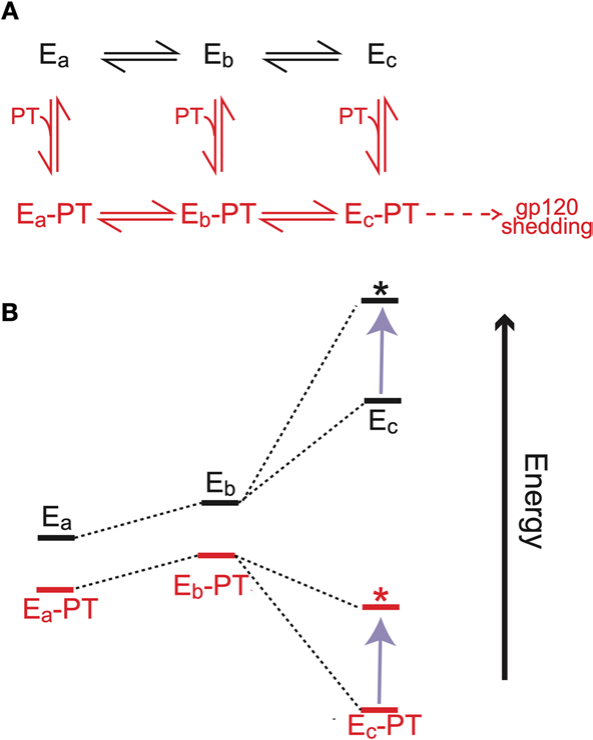
Proposed mechanism of PT escape. **A** Chemical reaction scheme depicting conformational fluctuations of unliganded (black) and PT-bound (red) Env in its prefusogenic state. **B** Energy levels of different conformations of unliganded and PT-bound Env. The effect of escape mutations on this energetic landscape is indicated by the purple arrow, where the asterisk represents the energy level of the mutated Env. Detailed descriptions of this model, including definitions of the different conformations, can be found in the text and Additional file 1: Figure S4

We have developed a simple working model of Env conformational transitions and ligand binding to account for the PT escape mechanism (Fig. 10; full explanation in legend to Additional file 1: Figure S4). In the model, Env is envisioned to exist in states E_a_, E_b_ and E_c_, representing different prefusogenic conformations of the Env trimer possibly related to states observed by single-molecule spectroscopy [41, 42]. Among these states, E_b_ and E_c_ are induced by CD4 binding but only E_c_ possesses a fully formed bridging sheet capable of interacting with mAb 17b or coreceptor. PTs can interact with all three states, but they preferentially stabilize E_c_, accounting for their ability to trigger gp120 shedding. We propose that PT escape mutations selectively destabilize the E_c_ conformation in both the unliganded and PT-bound states. As a consequence, PT-bound Env will fluctuate more into the E_a_ and E_b_ conformations, disrupting the affinity of PT for Env as well as reducing PT-induced gp120 shedding from the E_c_ conformation. The model also describes many of the features of CD4 and 17b interaction observed with the mutant Envs, including reduced CD4-induced gp120 shedding and the decreased 17b binding affinity due to a lower association rate (Additional file 1: Figure S4).

The escape profile observed in the current study must be borne in mind when considering future inhibitor design. A major benefit of the cPT class of entry inhibitors is the combination of attractive functions, such as irreversible Env inactivation, combined with conformational constraint enabling metabolic stability. This current study was the first attempt to ask how the virus would escape PTs. We were encouraged that resistance emerged significantly more slowly for PTs than for the high affinity fusion inhibitor C37 [24, 43, 44]; the observation suggests that PT escape does not arise easily. This delay might be tied to the indirect mechanism of resistance: rather than directly disrupting PT binding affinity, the escape mutations appear to modify stability of prefusogenic conformations along the fusion pathway. It remains to be seen whether such alteration in the Env energy landscape detrimentally affects HIV-1 replication fitness. Relevant to antiviral development, the allosteric nature of the resistance mechanism will provide a challenge for improving the cPT design, but fine tuning the pharmacophore to enhance its affinity for its preferred binding conformation will likely reduce the risk of viral escape. In addition, combining PTs with other entry inhibitors to form synergistically-acting non-covalent mixtures and covalent conjugates is always an option to help overcome viral escape [37, 39, 44].

## Conclusions

Peptide triazoles have been shown to irreversibly inactivate HIV-1 by triggering gp120 shedding and, in the case of peptide triazole thiols, by additionally inducing virion membrane disruption. Here, we showed that virus escape from both a macrocyclic PT and a linear thiol-containing variant with same pharmacophore occurred through similar dominant mutations both in and outside the inhibitor binding cavity, arguing for a similar escape route. The major escape mutants reflect the resistance mechanism through altering not only binding, but also the Env dynamic and conformational transitions. These mechanisms of escape provide mechanistic insights for HIV-1 inhibition by PT class of inhibitors and provide probes to direct future efforts for redesigning and improving the cyclic peptide triazole class of HIV-1 entry inhibitors.

## Materials and methods

### Reagents and viruses

HEK293T (HEK, human embryonic kidney cells) were purchased from ATCC (Manassas, VA). The following reagents were obtained from the NIH AIDS Reference and Reagent Program, Division of AIDS, NIAID: HOS CD4+ CXCR4+ Cells from Dr. Nathaniel Landau; pDOLHIVenv from Dr. Eric O. Freed and Dr. Rex Risser; TZM-bl cells from Dr. John C. Kappes, Dr. Xiayun Wu and Tranzyme Inc.; CEM-GFP cells from Dr. Jacques Corbeil; pNL4-3 from Dr. Malcom Martin; and monoclonal antibody 16H3 (ARP-12559) from Duke Human Vaccine Institute. The pNL4-3 Luc AM pseudotyping backbone plasmid was a generous gift from Dr. John Moore. Dr. Joseph Sodroski donated the expression plasmid for HIV-1 HxBc2 strain Envelope. *Escherichia coli* strains XL-10 gold (Agilent) and Stbl2 cells (Invitrogen) were used for propagating DNA. Thermostable DNA polymerase (PfuUltra™) was obtained from Stratagene Inc. (La Jolla, CA). Phusion® High-Fidelity Polymerase was purchased from New England Biolabs (Ipswich, MA). Integrated DNA Technologies supplied Custom-oligonucleotide primers. Polyethylenimine (PEI) 25 kDa linear polymer, was obtained from Polysciences, Inc. (Warrington, PA). All other reagents were purchased from Sigma-Aldrich unless otherwise specified.

### Peptide synthesis

A CEM microwave synthesizer (Liberty Blue) was used for solid phase peptide synthesis of PTs, as previously described [14, 21, 22, 39]. Fmoc-4-azido-Proline used in the synthesis was prepared as previously reported [21]. All other Fmoc-, Boc- protected amino acids, *N*,*N*ʹ-diisopropylcarbodiimide, ethyl (hydroxyimino) cyanoacetate (OxymaPure) and rink amide resin (100–200 mesh size, 0.53 meq/g substitution) were purchased from Chem-Impex International, INC. Ethynylferrocene and CuI catalyst for the click reaction, and hydrazine, were purchased from Sigma Aldrich. HPLC purifications were performed using a Waters® HPLC system with reverse phase (RP) C18 prep columns. Purity verification of cPTs was carried out by analytical C18 RP-HPLC column, using a BeckmanCoulter® HPLC system. HPLC grade acetonitrile (ACN), Millipore-MilliQ water and 0.1% TFA were used as solvents for the HPLC purification. Mass was confirmed using matrix-assisted laser desorption ionization time-of-flight mass spectrometry (MALDI-TOF MS).

### Passaging of fully-infectious virus with inhibitor dose escalation

Fully-infectious NL4-3 virus was used for virus passaging studies. Passaging in the presence of inhibitors was performed using a previously published protocol [45] and started at the determined IC_50_ value of the inhibitor. Culture supernatant was collected and lysed with Triton X-100, either weekly or biweekly, to determine the amount of p24 proteins present in the supernatant. The level of p24, determined by p24 ELISA [16, 23, 38] was dependent on the amount of virus able to be produced in the culture. When the level of p24 in peptide-treated cultures was within threefold comparable to the amount in untreated control cultures, the concentration of inhibitor in the culture was escalated two-fold. Three independent cultures for each treatment were carried out in 48-well plates using CEM-GFP cells [46] as the target cells for virus infection. Each plate only contained one treatment to prevent virus contamination between sample groups. Phosphate buffered saline (PBS) was also added to each plate between samples to provide a barrier between cultures and to prevent evaporation. Virus cultures were passaged each week by transferring 100 μL of culture supernatant onto 0.5 million fresh CEM-GFP cells in 500 μL of CEM medium (RPMI 1640 medium supplemented with 10% Fetal Bovine Serum, 100 unit/mL penicillin, 100 µg/mL streptomycin, 2 mM l-glutamine, and 500 μg/mL G418). After inoculating the culture each week, the remaining supernatant was aliquoted and frozen at − 80 °C. The cell pellet was then resuspended in PBS and frozen at − 80 °C in preparation for genomic DNA (gDNA) isolation.

### WST-1 cytotoxicity assay

The cytotoxicity was determined on CEM-GFP cells via a colorimetric assay to measure the relative proliferation rates of cells in culture. 7500 cells /well were seeded in 96-well plates and treated with varying concentrations of AAR029b and KR13 compounds at 37 °C. After 18 h of incubation, the media containing compounds were discarded and treated with 10% (v/v) WST-1 (a tetrazolium salt) in fresh media for one hour at 37 °C. The cell viability was determined by measuring the absorbance at 450 nm using a Tecan Infinite F50 plate reader.

### Neutralization assay with a fully-infectious virus strain

TZM-bl cells were seeded into 96-well plates at a density of 10,000 cells /well. After 24 h growth, HIV-1 strain fully-infectious NL4-3 was incubated with serial dilutions of inhibitors (AAR029b, KR13 or 17b) for 30 min at 37 °C before adding the mixture to the cells. The virus was allowed to infect target cells for 24 h. Cells were lysed using 1X passive lysis buffer (Promega). Three freeze–thaw cycles were conducted to help with cell lysis. 40 μL of cell lysate was transferred to a white 96-well plate (Greiner), mixed with 100 μL luciferin buffer (0.1 M potassium phosphate, 0.1 M magnesium sulfate, 0.1 M ATP and DTT) and 50 μL 1 mM D-luciferin (Anaspec). Luminescence was immediately measured using a Wallac 1450 Microbeta Luminescence reader at a wavelength of 490 nm. The data were analyzed in GraphPad Prism 9 using nonlinear regression fit with dose response-inhibitor equation (4 parameters) shown as below:

1 $$Y = {\text{Bottom }} + \frac{{\left( {{\text{Top}} - {\text{Bottom}}} \right)}}{{\left( {1 + \left( \frac{X}{IC50} \right)^{HillSlope} } \right)}}$$

Top and Bottom are the response values at high and low inhibitor concentrations, respectively. X and Y represent as the inhibitor concentrations and infection levels, respectively. IC_50_ is the concentration of inhibitor, at which gives a response halfway between Top and Bottom. The Hill Slope describes the slope of the sigmoidal curve between the Top and Bottom plateaus.

### Isolation and sequencing of the HIV-1 genome

Previously frozen cells for the desired week were taken out of the − 80 °C freezer and thawed. Genomic DNA from infected cells were obtained using the QIAamp DNA Mini and Blood Mini Kits (Qiagen) and following the manufacturer’s protocols. The HIV-1 Env genome was amplified using the specific five pairs of PCR primers. 1st: 5ʹ-CTATGGCAGGAAGAAGCGGAGAC-3ʹ and 5ʹ-ACCAGCCGGGGCACAATAATG-3ʹ; 2nd pair: 5ʹ-GCATGAGGATATAATCAGTTTA TGGG-3ʹ and 5ʹ-AATTCCCCTCCACAATTAAAACTG-3ʹ; 3rd pair: 5ʹ-GCTGTTAAATGGCA GTCTAGC-3ʹ and 5ʹ-CAAATGAGTTTTCCAGAGCAACCC-3ʹ; 4th pair: 5ʹ-GAGAAGAGTG GTGCAGAGAG-3ʹ and 5ʹ-CTATCTGTCCCCTCAGCTACTGC-3ʹ; 5th pair: 5ʹ-GATTGTGGA ACTTCTGGGAC-3ʹ and 5ʹ-TCCCAGGCTCAGATCTGGTCTAAC-3ʹ. Each primer set generated an approximately 1 kB fragment that overlapped with another PCR fragment to ensure full coverage of the Env genome. Amplification was accomplished using Phusion® High-Fidelity polymerase kit (New England Biolabs). The reaction for amplification was optimized as follows: 1 × High Fidelity buffer, 2.5 mM MgCl_2_, 0.35 mM dNTP mix, 0.3 mM of forward and reverse primers, and 0.5 units of Phusion® High-Fidelity polymerase. The reaction was initiated with 2 min of initial denaturation at 95 °C, followed by 40 cycles of amplification (94 °C for 30 s, 58 °C for 30 s, and 72 °C for 1 min), and a final elongation step of 72 °C for 10 min. PCR products were confirmed by agarose gel running, purified by using Wizard® PCR Clean-Up kit (Promega), and sequenced using Sanger Sequencing service provided by Genewiz, LLC.

### Site-directed mutagenesis of envelope expression plasmids

Site-directed mutagenesis were performed using a Quick-Change II XL Site-Directed Mutagenesis kit (Stratagene). Primers were designed and synthesized by IDT Inc. using solid-phase synthesis. Confirmed mutant plasmids were transformed into MAX Efficiency Stbl2 cells (Stratagene) to better support [23, 37–39] the retrovirus vector and produced in a large quantity using a Hispeed® Plasmid Maxiprep kit (Qiagen). Double mutations were prepared by conducting a second round of site-directed mutagenesis on the basis of the purified and confirmed DNA template. All the sequences of constructs were confirmed through Sanger sequencing done by Genewiz, LLC.

### Production and purification of envelope-pseudotyped virus

Neutralization assays were conducted with a luciferase reporter assay by using the single-round infection of envelope-pseudotyped viruses following a standard protocol [15, 21–23]. 8 μg envelope deficient provirus construct (pNL4-3.luc.AM [47]) with 4 μg Env expression vector (pDOLHIVenv /pHxBc2 Env) were co-transfected via a polyethyleneimine (PEI) transfection reagent into HEK293T cells. Supernatants were collected 48 to 72 h after transfection to obtain pseudotyped viruses. The supernatant with viral particles was spun down to remove cell pellet, filtered through a 0.45 μm polyethersulfone (PES) membrane to remove debris, and concentrated to 1.5 mL by a 100 kDa cut-off spin concentrator (Amicon Ultra Ultracell, Millipore). Gradient purification was performed with a continuous 6–20% iodixanol gradient. The concentrated virus supernatant was loaded on top of the 10 mL gradient. After a two-hour ultracentrifuge spin at 40,000 RPM at 4 °C, 10 fractions were tested for the presence of virus and the fractions 6–8 with viruses were confirmed and then pooled together. 0.3 mL aliquots were stored in -80 °C after fast freezing by dry ice. Purified viruses were validated by ELISA and infectivity assay.

### Neutralization assay with envelope-pseudotyped virus

HOS.T4.X4 cells were seeded at a density of 7500 cells per well in 96-well plates and cultured for 24 h at 37 °C. HIV-1 NL4-3 or HxBc2 pseudovirus corresponding to 500 ng p24 was incubated with serial dilutions of inhibitors (AAR029b, KR13 and 17b) for 30 min at 37 °C. The pseudovirus-inhibitor mixtures were loaded onto the seeded HOS.T4.X4 cells and incubated at 37 °C. Medium was changed after 24 h. Cell lysis was performed after another 24 h using 1X passive lysis buffer (Promega). Three freeze–thaw cycles were conducted to help with cell lysis. 40 μL of cell lysate was transferred to a white 96-well plate (Greiner), and mixed with 100 μL luciferin buffer (0.1 M potassium phosphate, 0.1 M magnesium sulfate, 0.1 M ATP and DTT) and 50 μL 1 mM D-luciferin (Anaspec). Luminescence was immediately measured using a Wallac 1450 Microbeta Luminescence reader at a wavelength of 490 nm. The data were analyzed in GraphPad Prism 9 using nonlinear regression fit with dose response-inhibitor equation (4 parameters).

### Protein expression and purification

The plasmid for HxBc2 gp120 wild type and mutants in pcDNA3.1(−) vector for transient transfection was purified using a ZymoPURE II Plasmid Maxiprep kit (Zymo Research) and transfected into HEK293F cells according to manufacturer’s protocol (Invitrogen). After five days of inoculation at 37 °C, cells were harvested and spun down. The supernatant was filtered through 0.2 μm filters. Purification was conducted over a 17b antibody-coupled column prepared using an NHS-activated Sepharose, HiTrap HP column (GE Healthcare). gp120 subunits were eluted by using 0.1 M glycine buffer (0.15 M NaCl, pH 2.4). The eluted protein was rapidly neutralized by the addition of 1 M Tris buffer (pH 8.0). Then buffer exchange was performed with 1 × PBS pH 7.4. The protein samples were applied to a HiLoad 26/60 Superdex 200 HR prepacked gel filtration column (GE). Purity of eluted fractions and monomeric state of HxBc2 gp120 were identified by SDS-PAGE/immunoblotting with mAb 16H3 (which recognize the C1 region of gp120). Monomeric gp120 were pooled, concentrated, frozen and stored in aliquots at − 80 °C.

### Thermodynamic profiles of binding by isothermal titration calorimetry (ITC) analysis

Equilibrium dissociation constants of AAR029b, KR13 and 17b IgG were determined at 25 °C on a VP-Isothermal Titration Calorimetry (VP-ITC) system (MicroCalTM, GE Healthcare, Freiburg). 60–100 µM of KR13, AAR029b or 17b in ITC Buffer (1 × PBS pH 7.4) were titrated in 8 µL steps into a reaction chamber containing 2–8 µM of HxBc2 gp120 WT, V255I and V255I/S143N monomers in the same buffer. The resulting heat changes upon injection were integrated over a time range of 240 s, and the obtained values were fit to a standard single site-binding model using Origin® software.

### Surface plasmon resonance (SPR) interaction analysis

SPR experiments were performed on a Biacore 3000 biosensor (Global Cytiva Lifesciences) at 25 °C using PBS-P (10 mM Phosphate, 150 mM NaCl, pH 7.4, 0.005% P-20) as the running buffer. A CM5 sensor chip was docked and derivatized by amine coupling with HIV-1_HxBc2_ WT and mutant gp120 subunits using freshly prepared 1:1 50 mM NHS (*N*-hydroxysuccinamide):200 mM EDC (1-ethyl-3-(3-(dimethylamino)propyl) carbodiimide). Flow cell 1 was left free to serve as a control for flow cells 2–4 containing 1800–2200 response units (RU) of HIV-1 HxBc2 gp120 WT, V255I and V255I/S143N monomers. Direct binding was determined by injecting AAR029b, KR13 or 17b IgG at concentrations spanning 0.20 nM to 3200 nM over all flow cells at a flow rate of 50 μL/min for 5 min (association phase) and all flow cell surfaces were washed with running buffer for 5 min (dissociation phase). The remaining bound analytes were removed with 1 pulse of 10 mM Glycine (pH 2.5) for 40 s. Next, soluble CD4 spanning 0.14 nM to 300 nM was injected over all flow cells at a flow rate of 50 μL/min with 5 min association phase and 10 min dissociation phase. Surface regeneration was attained using one pulse of 10 mM glycine (pH 2.5) for 40 s.

For data analysis, binding profiles were double referenced to minimize the impact of instrument and solvent noise. For AAR029b, 17b and CD4 interactions, the sensorgrams from two data sets were globally fit using Scrubber (BioLogic Software) to a Langmuir 1:1 binding model to obtain second-order association rate constant k_1_ and first-order dissociation rate constant k_−1_. The equilibrium dissociation constant was calculated as K_D_ = k_−1_/k_1_. For KR13 interactions, the sensorgrams were fit to a three-state binding/isomerization model as depicted below:

2 $${\text{Env }}\begin{array}{*{20}c} {{\text{k}}_{1} \left[ {{\text{PT}}} \right]} \\ \rightleftharpoons \\ {{\text{k}}_{ - 1} } \\ \end{array} {\text{ Env}} \cdot {\text{PT }}\begin{array}{*{20}c} {{\text{k}}_{2} } \\ \rightleftharpoons \\ {{\text{k}}_{ - 2} } \\ \end{array} {\text{ Env}} \cdot {\text{PT}}^{*}$$

The model depicts a biomolecular interaction between KR13 (here depicted as PT) and Env followed by a hypothesized disulfide rearrangement induced by the inhibitor thiol. Rate constants k_1_ and k_-1_ are defined as they are in the 1:1 binding model, while k_2_ and k_-2_ are first order rate constants describing isomerization and de-isomerization, respectively. Equations describing the time course of the interactions can be found in Additional file 1: Method S1. For the fit, it was assumed that the states $\mathrm{Env}\cdot \mathrm{PT}$ and ${\mathrm{Env}\cdot \mathrm{PT}}^{*}$ had the same SPR signal. Rate constants were determined using a least-squares gradient-step algorithm coded in Visual Basic and implemented through Microsoft Excel. The KR13 equilibrium dissociation constant was calculated as:

3 $$K_{D} = \frac{{k_{ - 1} k_{ - 2} }}{{k_{1} \left( {k_{2} + k_{ - 2} } \right)}}$$

### Molecular modeling of the peptide triazole binding to a fully-glycosylated HIV-1 trimer

Preparation and molecular docking were performed using an established methodology [14]. Briefly, peptides (UM15 and AAR029b) and protein (chain C of the Env trimer PDB code 5FUU [9] were prepared for the docking study in silico by using The Schrödinger package (Schrödinger Suite 2014; Schrödinger, LLC) and by Autodock tools graphical interface (MGtools 1.5.6rc3). These minimized and repaired structures were saved as PDB files for use in the docking simulations. Flexible docking was carried out using Autodock and setting Trp112 and Trp427 as flexible. Cluster analysis was performed on docked results, with a root-mean-square tolerance of 2.0 Å. Visual inspection of the lower energy docked poses was compared to the prior modeling work with the monomeric gp120 [15]. Considering the mutagenesis analysis results, the lowest binding energy pose that matched the mutational and structure–activity analyses [15] was selected as a representative complex for each compound. Molecular graphics and steric clash analyses were performed with the UCSF Chimera package. Chimera is developed by the Resource for Biocomputing, Visualization, and Informatics at the University of California, San Francisco (supported by NIGMS P41-GM103311) [48].

### Determination of CD4 utilization in viral entry

Neutralization assay was performed as described previously by inhibiting HIV-1 recombinant virus NL4-3 infection with D23.2 (DARPin 23.2 [36]). The infectivity levels of HIV-1_NL4-3_ WT and mutants (S143N, V255I, V255I/S143N) were determined in the presence of D23.2 (11 nM, about IC_50_ value). We displayed the absolute infectivity in the presence and absence of D23.2. The relative infectivity (+D23.2/−D23.2) was plotted to better indicate the CD4 utilization in the WT and mutant recombinant viruses.

### gp120 shedding analysis

The gp120 shedding assay was performed as described previously [49]. Briefly, three million HEK293T cells per flask were transiently transfected with 4 μg of HIV-1 NL4-3 Env plasmid and transfection reagent FuGENE 6 (Promega). 5 mM EDTA in DPBS was used to detach cells at 24 h and cells were reseeded in 96-well plates with 50,000 cells per well. Mock and transfected HEK293T were treated with serial dilutions of sCD4 183, AAR029b or a PBS control for 2 h at 37 °C. Cells were washed with fresh medium, detached with 5 mM EDTA, and then washed and resuspended in FC buffer (1% BSA and 1 mM EDTA in 10 mM PBS pH 7.4). 2% paraformaldehyde in DPBS was used to fix the cells for 15 min at room temperature. Cells were then washed and resuspended in the FC buffer three times. The fixed cells were stained with 5 μg/mL antibody 35O22 (NIH AIDS Reagent Program) for one hour at room temperature and washed three times with FC buffer. Secondary stain anti-Human PerCP in 1:500 dilution (Jackson ImmunoResearch Laboratories, Inc., West Grove, PA) was incubated with cells for one hour at room temperature, and washed three times with FC buffer. Cell counts and Env staining were assessed in counted cells on a Guava EasyCyte 5HT flow cytometry system (Millipore). Analyzed cells were subjected to forward/side scatter gating based on untreated control populations of identical transfection, and median fluorescence values were obtained using Guava InCyte 3.2 software. Data were analyzed in GraphPad Prism 9 using nonlinear regression fit with dose response-inhibitor equation (4 parameters).

## Supplementary Information

**Additional file 1: Figure S1.** Cytotoxicity assessment of AAR029b and KR13. **Figure S2.** Aligned Env sequences obtained from viral cultures propagated in the absence (NC) or presence of AAR029b or KR13. **Figure S3.** Biacore 3000 sensorgrams for sCD4 binding to wild type and resistant gp120. **Figure S4.** Working model of the effect that PT escape mutations have on Env conformational fluctuations and the binding/inhibition of CD4 and 17b. **Method S1.** SPR fitting model with KR13 binding with Env gp120.

## Data Availability

The datasets used and/or analyzed during the current study are available from the corresponding author on reasonable request.

## Abbreviations

HIV-1: Human immunodeficiency virus type 1
PT: Peptide triazole
cPT: Cyclic peptide triazole
Env: Envelope protein
HEK: Human Embryonic Kidney cells
HOS: Human Osteosarcoma cells
PEI: Polyethylenimine
WT: Wild-type
SPR: Surface plasmon resonance
ITC: Isothermal titration calorimetry
T4: CD4
X4: CXCR4
