# Supplementary material for: Altered Env conformational dynamics as a mechanism of resistance to peptide-triazole HIV-1 inactivators
Source: Retrovirology. 2021 Oct 9;18:31. doi: 10.1186/s12977-021-00575-z (PMC8501640; doi:10.1186/s12977-021-00575-z)
Supplement: Supplementary file 1 — Additional file 1: Figure S1. Cytotoxicity assessment of AAR029b and KR13. Figure S2. Aligned Env sequences obtained from viral cultures propagated in the absence (NC) or presence of AAR029b or KR13. Figure S3. Biacore 3000 sensorgrams for sCD4 binding to wild type and resistant gp120. Figure S4. Working model of the effect that PT escape mutations have on Env conformational fluctuations and the binding/inhibition of CD4 and 17b. Method S1. SPR fitting model with KR13 binding with Env gp120. [file 12977_2021_575_MOESM1_ESM.docx]

**Additional File**

**
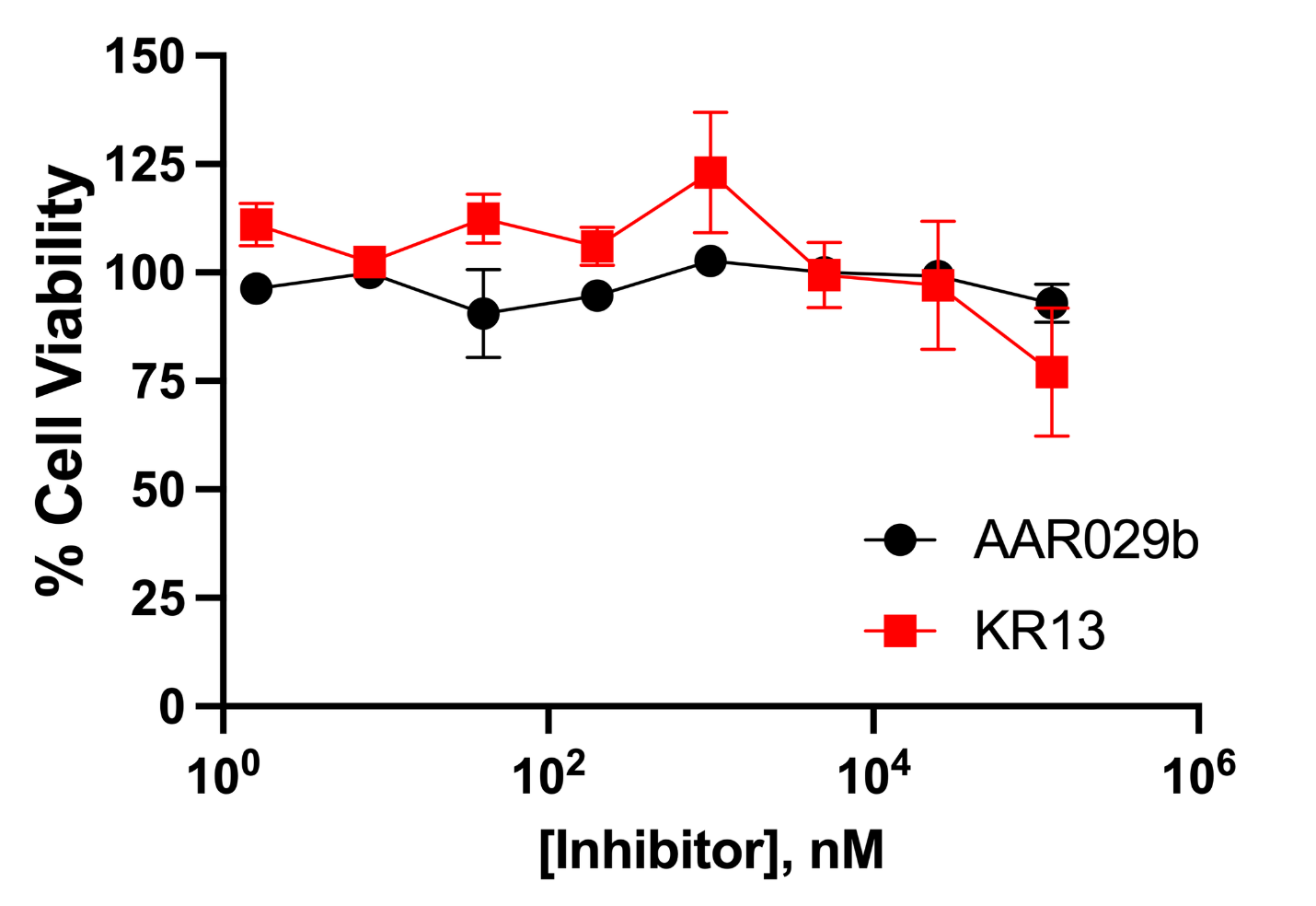
**

**Figure S1. Cytotoxicity assessment of AAR029b and KR13.** CEM-GFP cells were incubated in increasing concentrations of AAR029b (black) and KR13 (red), and cell viability was measured by WST-1 assay. For KR13, cytotoxicity was noted for concentrations above 25 μM, with ~30% of cells dying at a concentration of 125 μM. For AAR029b, cytotoxicity was minimal even up to concentrations of 125 μM. Data represent means and standard deviations of three independent experiments performed in triplicate.

**Figure S2. Aligned Env sequences obtained from viral cultures propagated in the absence (NC) or presence of AAR029b or KR13. A. 6 weeks. B. 10 weeks. C. 15 weeks.**

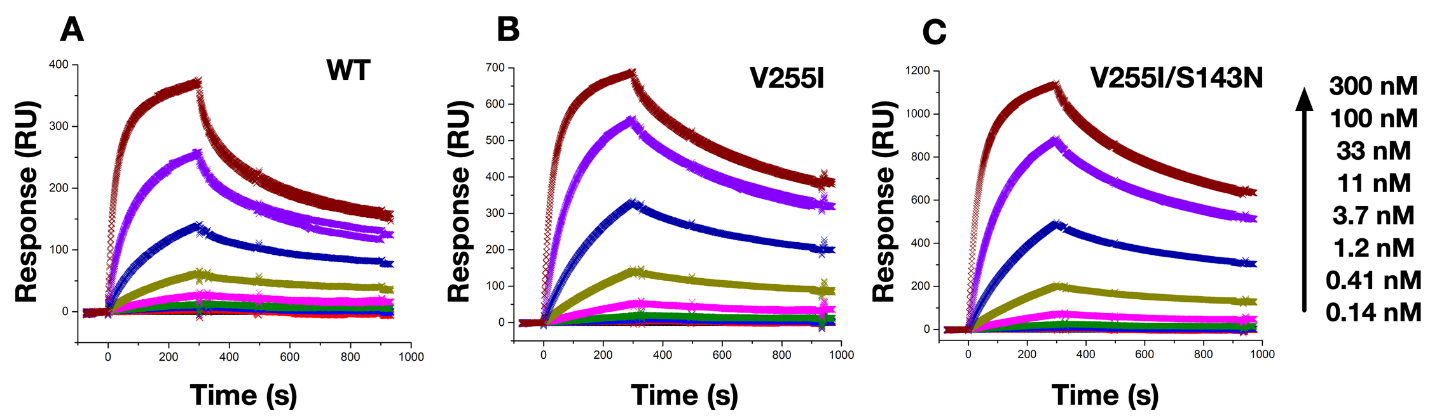


**Figure S3. Biacore 3000 sensorgrams for sCD4 binding to wild type and resistant gp120**. Serial 3-fold dilutions of sCD4 starting at 300 nM were flowed over immobilized monomeric HxBc2 gp120 variants: (A) wild type, (B) V255I and (C) V255I/S143N. Two replicates are shown to illustrate the adequacy of our regeneration method. Each sensogram set was fit globally to a 1:1 Langmuir binding model to derive the kinetic parameters summarized in **Fig. 8A**.

**
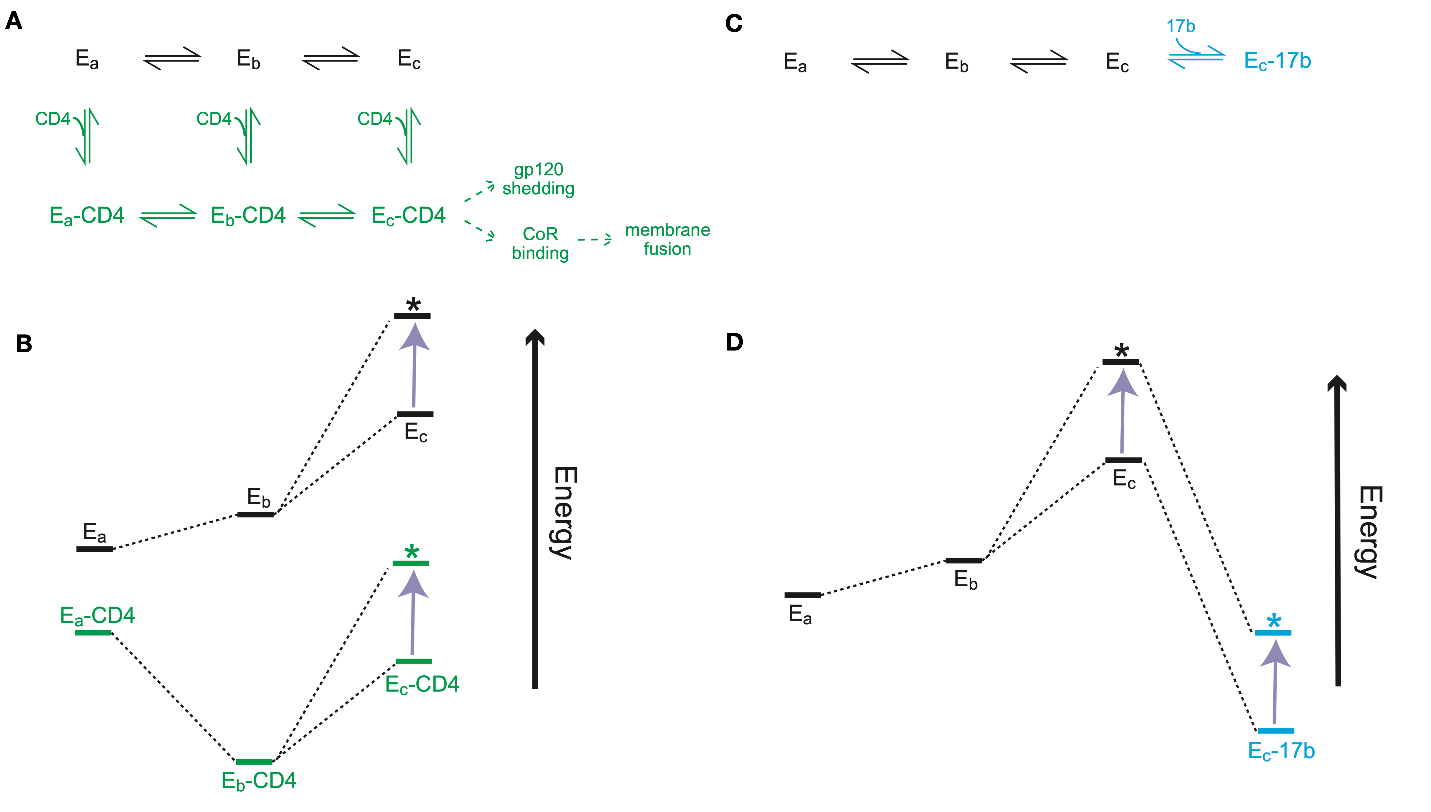
**

**Figure S4: Working model of the effect that PT escape mutations have on Env conformational fluctuations and the binding/inhibition of CD4 and 17b. (Explanation in conjunction with Fig. 10 of main text working model for resistance.) A and C.** Chemical reaction schemes depicting conformational fluctuations of unliganded (black) and CD4-bound (green in **A**) or 17b-bound (blue in **C**) Env in its prefusogenic state. **B and D**. Energy levels of different conformations of unliganded, CD4-bound (**B**) and 17b-bound (**D**) Env.

E_a_, E_b_ and E_c_ represent different prefusion conformations of Env, with transition from E_a_ to E_c_ being on pathway to Env activation and membrane fusion. E_b_ and E_c_ represent CD4-stabilized conformations, but only E_c_ adopts the structure required to bind CoR or mAb 17b. PT can bind to any of the three conformations, but preferentially stabilizes E_c_ (**Fig. 10A**). In the unliganded state, E_c_ is substantially higher in energy than E_a_ and E_b_, as required to restrict CD4-independent entry in the presence of non-infectible cells or spontaneous gp120 shedding in their absence (**Fig. 10B**). In the PT-bound state, the E_c_ energy is substantially lowered, accounting for the ability of PT to trigger gp120 shedding. In our model, PT escape mutations specifically destabilize the E_c_ conformation in both the unliganded and PT-bound states (purple arrow). As E_c_ is sparsely populated in the unliganded state, the mutations do not significantly impact the structure and dynamics of unliganded Env. However, due to destabilization of PT-bound E_c_, its energy is brought closer to the levels of PT-bound E_a_ and E_b_. As a consequence, the conformational equilibrium between PT-bound states is shifted from primarily E_c_ in wild type Env to a mixture of E_a_, E_b_ and E_c_ in resistant Env. This shift has three main outcomes relevant to PT binding. First, increased fluctuations into E_a_ and E_b_ raise the conformational disorder of the bound state, decreasing the entropic penalty of PT association. Second, spending less time, on average, in E_c_ leads to an overall decrease in favorable PT bonding interactions, reducing the enthalpy released upon PT association. Third, having fewer favorable bonding interactions on average increases dissociation kinetics (note: the association rate remains unchanged since the mutations have minimal impact on the distribution among E_a_, E_b_ and E_c_ in unliganded Env). These effects on PT binding work in concert to depopulate the E_c_ conformation, thereby hampering the ability of PT to irreversibly inactivate Env through gp120 shedding.

The PT working model also accurately predicts properties of the Env-CD4 interaction (**Figure S4 A-B**). CD4 binding preferentially stabilizes E_b_ and E_c_, increasing the likelihood of transitioning to the E_c_ state and undergoing gp120 shedding in the absence of appropriate target cells or CoR-induced membrane fusion in their presence. As with PT resistance, destabilizing E_c_ reduces fluctuations into that conformation, thereby hindering CD4-induced gp120 shedding. Unlike PT binding, however, destabilizing E_c_ does not substantially modify the distribution among CD4-bound Env conformations (primarily E_b_) and, thus, minimally impacts CD4 dissociation kinetics. Since CD4 association kinetics are also largely unaffected by escape mutations (as described above for PT association), CD4 binding affinity is the same for wild type and resistant Envs.

The model also accurately predicts some of the key interaction and inhibition properties of mAb 17b (**Figure S4 C-D**). Since the mAb interacts only with the E_c_ conformation, the 17b association rate critically depends on the relative amount of time spent in that one conformation. Hence, destabilization of E_c_ in resistant Envs directly reduces 17b association kinetics, thereby resulting in reduced binding affinity and inhibitor potency (note: dissociation kinetics should not be affected by escape mutations as this reaction only involves Env in its E_c_ conformation). However, the model fails to fully describe 17b thermodynamic data, probably because Env fluctuates between more than three conformational states. In fact, the observed PT-induced disruption of 17b binding [1,2], despite the two inhibitors having different binding sites, supports the existence of additional CD4-induced states before or after E_c_.

**Method S1. SPR fitting model with KR13 binding with Env gp120**

The sensorgrams for KR13 interactions with gp120 were analyzed using a three-state equilibrium binding model as shown in (1). In this model, KR13 (denoted as PT) binds to Env gp120 (denoted as Env) to form an intermediate $Env\cdot PT$, and then the complex undergoes disulfide exchange induced by the thiol group of KR13, transforming into the ${Env\cdot PT}^{*}$ state:

$\mathrm{Env}\begin{matrix} k_{1}[PT] \\ \rightleftharpoons\\ k_{-1} \end{matrix} Env\cdot PT \begin{matrix} k_{2} \\ \rightleftharpoons\\ k_{-2} \end{matrix}{Env\cdot PT}^{*}$ (1)

Association rate constant k_1_ has units of M^-1^ s^-1^, while and dissociation rate constant k_-1_  has units of s^-1^. Isomerization rate constant k_2_ and de-isomerization rate constant k_-2_ have units of s^-1^. By the law of mass action, the equilibrium probabilities of the Env ($E_{\infty}$), $Env\cdot PT$ (${EP}_{\infty}$) and ${Env\cdot PT}^{*}$ (${EP}_{\infty}^{*}$) are defined as:

$E_{\infty}=\frac{k_{-1}k_{-2}}{k_{-1}k_{-2}+k_{1}{[PT]k}_{2}+k_{1}[PT]k_{-2}}$ (2)

${EP}_{\infty}=\frac{k_{1}[PT]k_{-2}}{k_{-1}k_{-2}+k_{1}{[PT]k}_{2}+k_{1}[PT]k_{-2}}$ (3)

${EP}_{\infty}^{*}=\frac{k_{1}[PT]k_{2}}{k_{-1}k_{-2}+k_{1}{[PT]k}_{2}+k_{1}[PT]k_{-2}}$ (4)

where [PT] is the concentration of KR13. The equilibrium dissociation constant can be calculated as:

$K_{D}=\frac{E_{\infty}[PT]}{{EP}_{\infty}+{EP}_{\infty}^{*}}=\frac{k_{-1}k_{-2}}{k_{1}(k_{2}{+k}_{-2})}$ (5)

We define [E](t), [EP](t) and [EP^*^](t) as the time-dependent probability of unbound gp120 (Env), non-covalently-bonded gp120-KR13 complex ($Env\cdot PT$) and covalently-bonded gp120-KR13, respectively. The time course of these probabilities can be represented as

$\left[ E \right]\left( t \right)=A_{1}e^{\lambda_{+}t}+ B_{1}e^{\lambda_{-}t}+ E_{\infty}$ (6)

$\left[ EP \right]\left( t \right)=A_{2}e^{\lambda_{+}t}+ B_{2}e^{\lambda_{-}t}+ {EP}_{\infty}$ (4)

$\left[ {EP}^{*} \right]\left( t \right)=A_{3}e^{\lambda_{+}t}+ B_{3}e^{\lambda_{-}t}+ {EP}_{\infty}^{*}$ (7)

where A_i_, B_i_ and $\lambda_{\pm}$ are time-independent constants that depend on the rate constants, [PT], the starting probabilities of each state ($E_{0}, {EP}_{0}, {EP}_{0}^{*})$ and the equilibrium probabilities of each state ($E_{\infty}, {EP}_{\infty}, {EP}_{\infty}^{*}$). These constants can be derived from eigenvalue decomposition and take on the following values:

$\lambda_{\pm}=-\frac{1}{2}\left( k_{1}\left[ P \right]+k_{-1}+k_{2}+k_{-2} \right)\pm\frac{1}{2}\sqrt{\left( k_{1}\left[ P \right]+k_{-1}+k_{2}+k_{-2} \right)^{2}-4(k_{1}\left[ P \right]k_{2}+k_{1}\left[ P \right]k_{-2}+k_{-1}k_{-2}})$ (8)

$A_{1}=\left( \frac{k_{1}[P]+\lambda_{-}}{\lambda_{-}-\lambda_{+}} \right)\times\left[ E_{0}-E_{\infty}-\frac{k_{-1}\left( {EP}_{0}-{EP}_{\infty} \right)}{k_{1}\left[ P \right]+\lambda_{-}} \right]$ (9)

$A_{2}=\left( \frac{k_{1}[P]+\lambda_{+}}{k_{-1}} \right)\times A_{1}$ (10)

$A_{3}=\frac{k_{2}}{k_{-1}}\times\left( \frac{k_{1}\left[ P \right]+\lambda_{+}}{k_{-2}+\lambda_{+}} \right)\times A_{1}$ (11)

$B_{1}=k_{-1}\left( \frac{{EP}_{0}-{EP}_{\infty}}{\lambda_{-}-\lambda_{+}} \right)-\left( k_{1}\left[ P \right]+\lambda_{+} \right)\left( \frac{E_{0}-E_{\infty}}{\lambda_{-}+\lambda_{+}} \right)$ (12)

$B_{2}=\left( \frac{k_{1}\left[ P \right]+\lambda_{-}}{k_{-1}} \right)\times B_{1}$ (13)

$B_{3}=\frac{k_{2}}{k_{-1}}\left( \frac{k_{1}\left[ P \right]+\lambda_{-}}{k_{-2}+\lambda_{-}} \right)\times B_{1}$ (14)

**References:**

1. Bailey LD, Kalyana Sundaram RV, Li H, Duffy C, Aneja R, Rosemary Bastian A, et al. Disulfide Sensitivity in the Env Protein Underlies Lytic Inactivation of HIV-1 by Peptide Triazole Thiols. ACS Chem Biol. 2015;10: 2861–2873. doi:10.1021/acschembio.5b00381

2. Rashad AA, Kalyana Sundaram RV, Aneja R, Duffy C, Chaiken I. Macrocyclic Envelope Glycoprotein Antagonists that Irreversibly Inactivate HIV-1 before Host Cell Encounter. J Med Chem. 2015;58: 7603–7608. doi:10.1021/acs.jmedchem.5b00935
